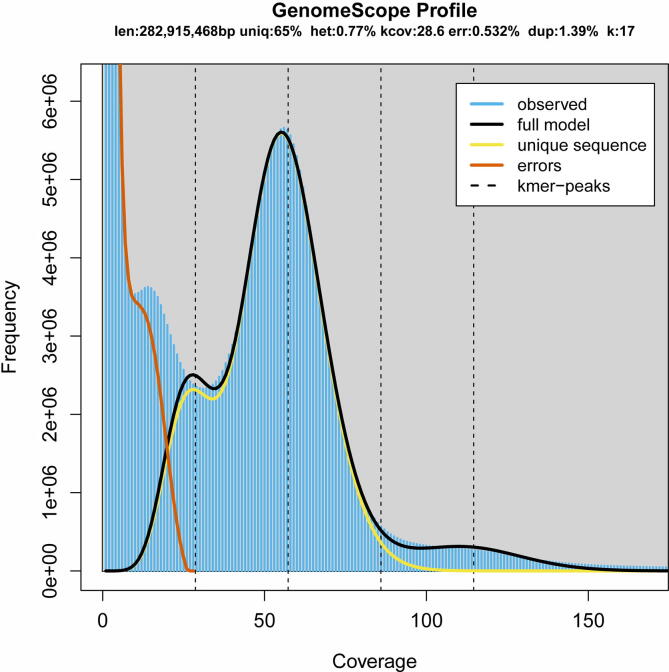# Genome of the Giant Panda Roundworm Illuminates Its Host Shift and Parasitic Adaptation

**DOI:** 10.1016/j.gpb.2021.08.002

**Published:** 2021-09-03

**Authors:** Yue Xie, Sen Wang, Shuangyang Wu, Shenghan Gao, Qingshu Meng, Chengdong Wang, Jingchao Lan, Li Luo, Xuan Zhou, Jing Xu, Xiaobin Gu, Ran He, Zijiang Yang, Xuerong Peng, Songnian Hu, Guangyou Yang

**Affiliations:** 1 Department of Parasitology, College of Veterinary Medicine, Sichuan Agricultural University, Chengdu 611130, China; 2 Agricultural Genomics Institute, Chinese Academy of Agricultural Sciences, Shenzhen 518120, China; 3 State Key Laboratory of Microbial Resources, Institute of Microbiology, Chinese Academy of Sciences, Beijing 100101, China; 4 Department of Oncology and Pathology, Karolinska Institutet, Stockholm 17164, Sweden; 5 Department of Urology, Feinberg School of Medicine, Northwestern University, Chicago, IL 60611, USA; 6 Chengdu Research Base of Giant Panda Breeding, Chengdu 610081, China; 7 Institute of Animal Genetics and Breeding, College of Animal Science and Technology, Sichuan Agricultural University, Chengdu 611130, China; 8 Department of Civil and Environmental Engineering, University of Maryland, College Park, MD 20740, USA; 9 Department of Chemistry, College of Life and Basic Science, Sichuan Agricultural University, Chengdu 611130, China; 10 University of Chinese Academy of Sciences, Beijing 100049, China

**Keywords:** Giant panda, *Baylisascaris* genome, Host shift, Parasitism evolution, Host adaptation

## Abstract

*Baylisascaris schroederi*, a roundworm (ascaridoid) parasite specific to the bamboo-feeding **giant panda** (*Ailuropoda melanoleuca*), represents a leading cause of mortality in wild giant panda populations. Here, we present a 293-megabase chromosome-level genome assembly of *B. schroederi* to infer its biology, including **host adaptations**. Comparative genomics revealed an evolutionary trajectory accompanied by host-shift events in ascaridoid parasite lineages after host separations, suggesting their potential for transmission and rapid adaptation to new hosts. Genomic and anatomical lines of evidence, including expansion and positive selection of genes related to the cuticle and basal metabolisms, indicate that *B. schroederi* undergoes specific adaptations to survive in the sharp-edged bamboo-enriched gut of giant pandas by structurally increasing its cuticle thickness and efficiently utilizing host nutrients through gut parasitism. Additionally, we characterized the secretome of *B. schroederi* and predicted potential drug and vaccine targets for new control strategies. Overall, this genome resource provides new insights into the host adaptation of *B. schroederi* to the giant panda as well as the host-shift events in ascaridoid parasite lineages. Our findings on the unique biology of *B. schroederi* will also aid in the development of prevention and treatment measures to protect giant panda populations from roundworm parasitism.

## Introduction

The giant panda (*Ailuropoda melanoleuca*) is an enigmatic and endangered mammalian species endemic to Western China. Unlike other bear members in Ursidae, which are carnivores or omnivores, the giant panda almost exclusively feeds on highly fibrous bamboo, despite its carnivoran alimentary tract [1], [2]. Consequently, giant pandas exhibit very low digestive efficiency and metabolic rates to maintain their daily energy balance [2]. Based on this physiological situation, the alimentary tract of giant pandas likely exhibits reduced nutrient digestibility and absorption, and is full of undigested and sharp-edged bamboo culms/branches. The former speculation explains why they spend most of each day consuming a remarkable quantity of bamboo relative to their body size [1], and the latter illustrates potential risks to both the gastrointestinal system (*e.g.*, physical damage to the stomach and gut) and tract-inhabiting organisms, including parasitic nematodes (*e.g.*, physical pressure and damage to worm bodies) [3].

**Supplementary Figure S1:**
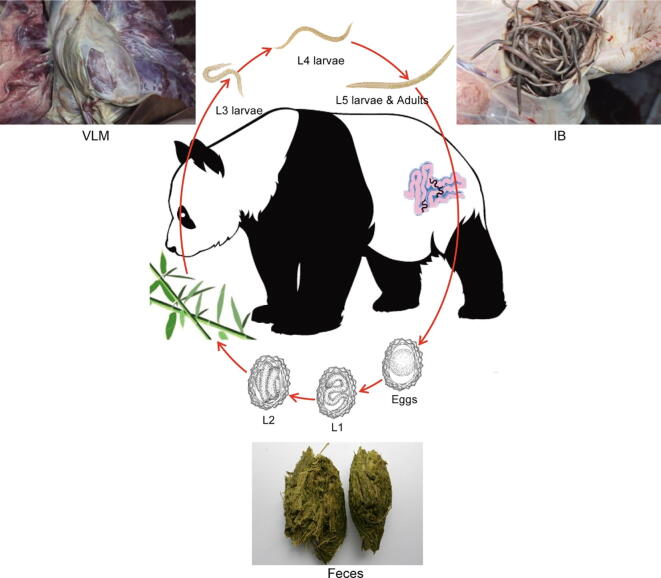


The roundworm *Baylisascaris schroederi* is the only endoparasite consistently found in giant pandas; it has been confirmed to be a leading cause of death in wild populations [4], [5]. *B. schroederi* infection in giant pandas follows a trophic pathway from ingestion to lifecycle completion without intermediate hosts (Figure S1). The adult *B. schroederi* inhabits the intestines of giant pandas and can cause intestinal obstruction, inflammation, and even host death. In addition, the larvae can disseminate into various body tissues and induce extensive inflammation and scarring of the intestinal wall, as well as the parenchyma of the liver and lung (also known as visceral larva migrans) [3], [4], [5]. Parasitological evidence shows that the adaptation of *B. schroederi* to its host is highly evolved, even compared to that of other ascaridoid parasites, including *Ascaris suum* in pigs and *Parascaris univalens* in horses [6]. Such physical adaptations to the nutrient-limited and fiber-enriched intestinal environment of giant pandas are likely related to nutritional metabolic changes and exoskeletal cuticle resistance. However, the detailed molecular mechanisms underlying these adaptation processes remain unknown.

To address these concerns and strengthen efforts to control infection in giant pandas, we generated a 293-megabase (Mb) chromosome-level genome assembly of *B. schroederi* and compared it with those of other ascaridoid species. The analysis identified a total of 16,072 nonredundant protein-coding genes in *B. schroederi*, and comparative genomics revealed the potential host shift among ascaridoid parasites and the coevolution of these species. During its parasitism process, *B. schroederi* appears to have evolved a thicker cuticle against the harsh intestinal environment and specialized its metabolic pathways to better utilize the limited nutrients during parasitism in the giant panda gut. Moreover, the enriched proteases in *B. schroederi* are linked to potential roles in host evasion and immunoregulation. Our findings provide a useful resource applicable to a wide range of fundamental biological studies of *Baylisascaris* and will strengthen the development of treatment and prevention options, enhancing giant panda conservation efforts.

## Results

### A high-quality, chromosome-level reference genome of *B. schroederi*

**Figure 1:**
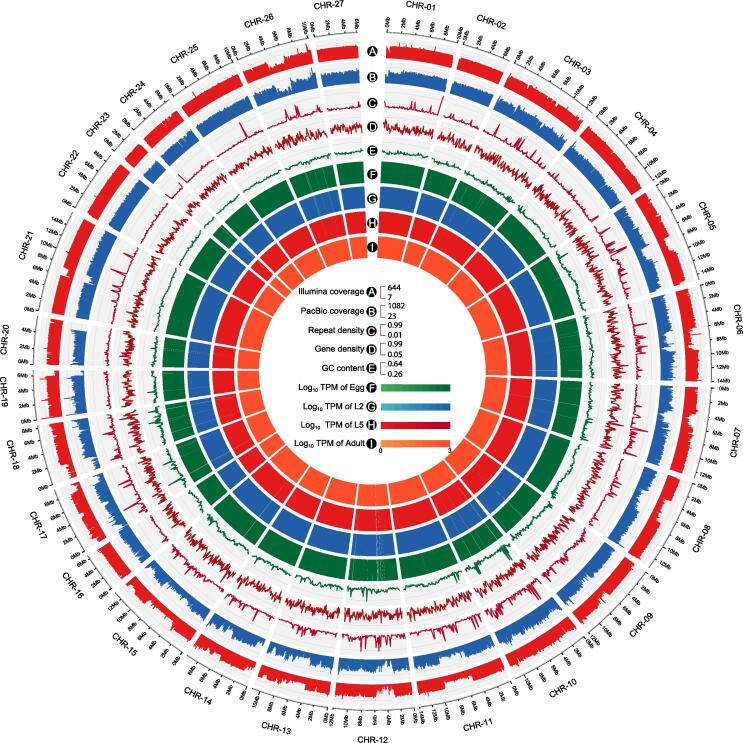
**Genomic features of the *Baylisascaris schroederi* genome** The rings depict the following information with a window size of 100 kb. **A.** Illumina sequencing coverage. **B.** PacBio sequencing coverage. **C.** Repeat density. **D.** Gene density. **E.** GC content. **F.**–**I.** Gene expression levels at the Egg (F), L2 (G), L5 (H), and Adult (I) stages of *B. schroederi*. Egg, embryonated eggs; L2, the second-stage larvae; L5, the intestine-inhabited fifth-stage larvae; Adult, the female adults.

**Supplementary Figure S2:**
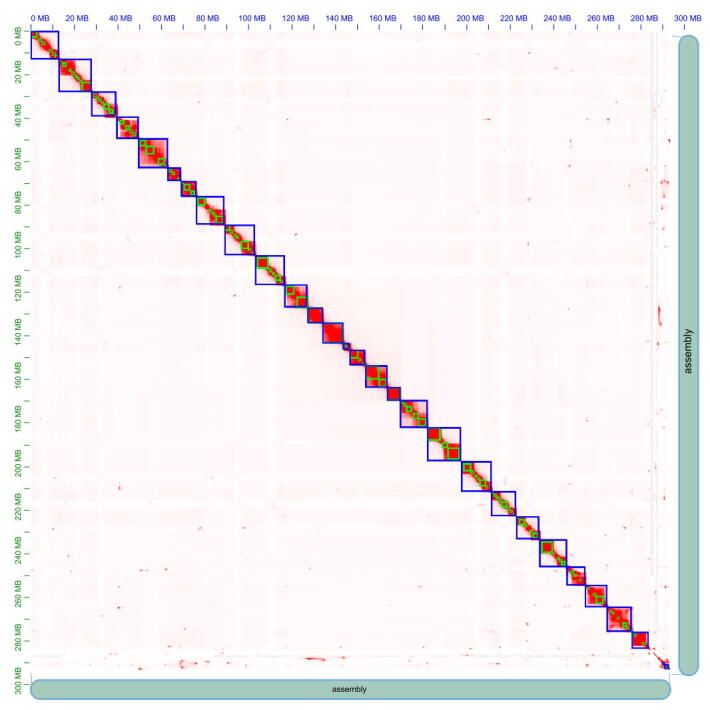


**Supplementary Figure S3:**
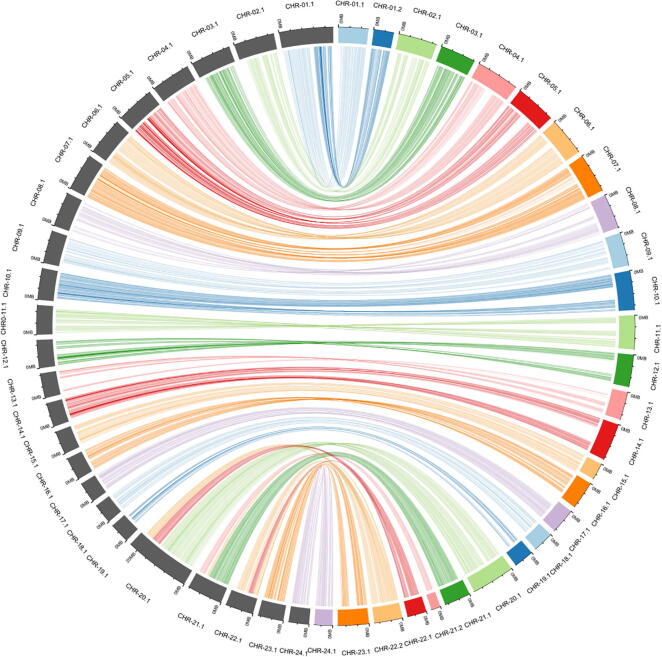


**Supplementary Figure S4:**
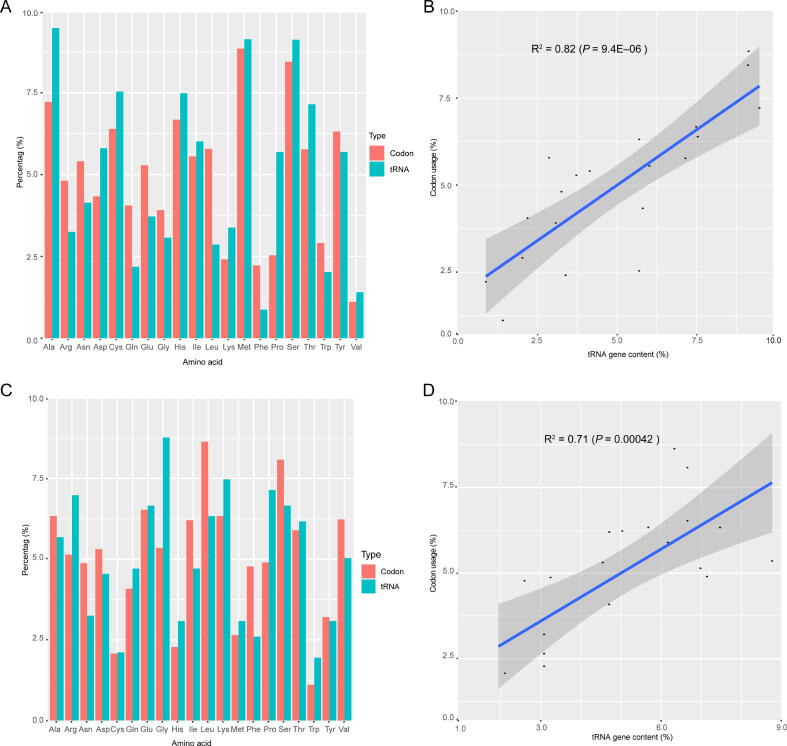


Using a combination of Illumina whole-genome shotgun technology, PacBio single-molecule real-time (SMRT) sequencing, and Hi-C scaffolding (Table S1), we produced a high-quality, chromosome-level reference genome of *B. schroederi*. The size of the assembled genome was 293 Mb with a contig N50 size of 1.92 Mb and a scaffold N50 size of 11.8 Mb, and was anchored to 27 chromosome-level pseudomolecules (Figure 1, Figures S2 and S3; Table 1, Table S2). The assembly size was larger than that of the horse parasite *P. univalens* (253 Mb) [7], comparable to that of the pig parasite *A. suum* (298 Mb) [8] and smaller than that of the dog parasite *Toxocara canis* (317 Mb) [9]. The GC content of the assembly was 37.6%, which was similar to that of *A. suum* (37.8%) but slightly lower than those of the *P. univalens* (39.1%) and *T. canis* (39.9%) assemblies. The completeness of the *B. schroederi* genome was estimated to achieve 97.9% (961/982) coverage using the core Benchmarking Universal Single-Copy Ortholog (BUSCO) genes and 91.2% mapping rate using the transcriptomic data from four stages across the lifecycle: embryonated eggs (Egg), the second-stage larvae (L2), the intestine-inhabited fifth-stage larvae (L5), and adult females (Adult). This confirmed that the assembly represented a substantial proportion of the entire genome (Table 1, Tables S3 and S4). The *B. schroederi* genome contained 12.0% repetitive sequences, equal to 35.3 Mb of the assembly, and these included 0.49% DNA transposons, 2.9% retrotransposons, 5.8% unclassified dispersed elements, and 2.6% simple repeats (Table S5). Moreover, 6190 transfer RNA (tRNA) and 978 ribosomal RNA (rRNA) genes were identified in the assembled genome, and the copy numbers reflected their codon usage in protein-coding regions (Figure S4; Tables S6 and S7; File S1).

**Table 1:** **Statistics of the genome features of four ascaridoid species**

	***Baylisascarisschroederi***	***Ascarissuum***	***Parascarisunivalens***	***Toxocaracanis***
Version	BSFv2.0	ASM18702v3	ASM225920v1	ADULT_r1.0
Genome size (bp)	293,522,654	298,028,455	253,353,821	317,115,901
Number of scaffolds	150	415	1274	22,857
Scaffold N50 size (Mb)	11.8	4.6	1.8	0.4
GC content (%)	37.6	37.8	39.1	39.9
Repeat (%)	12.0	11.1	7.7	12.9
Number of protein-coding genes	16,072	16,778	14,325	18,596
Gene density (number/Mb)	54.7	56.3	56.5	58.6
Mean protein length (aa)	492	436	470	385
BUSCO (complete/fragment, %)	92.2/5.7	89.1/7.2	91.0/5.8	87.0/8.0

*Note*: BUSCO, Benchmarking Universal Single-Copy Ortholog; aa, amino acid.

**Supplementary Figure S5:**
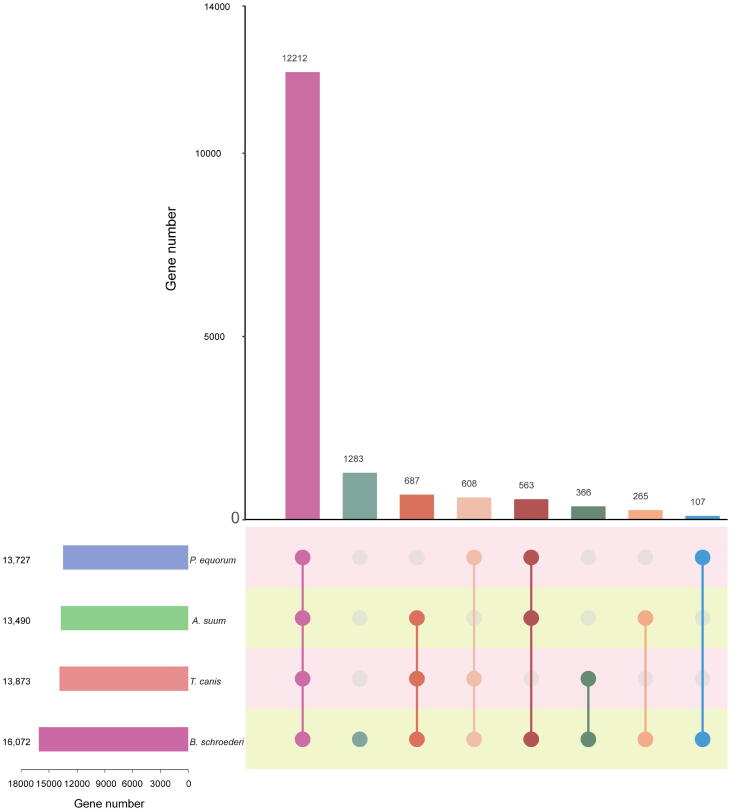


*De novo* predictions, homology-based searching, and deep transcriptome sequencing at multiple lifecycle stages (Egg, L2, L5, and Adult) identified a total of 16,072 protein-coding genes with an average length of 9452 bp, an exon length of 155 bp, and the estimated 9.5 exons per gene (Table S8), which were comparable to the data obtained for *A. suum*, *P. univalens*, and *T. canis* [7], [8], [9]. In addition, 90.6% of the gene set was supported by the mapping of RNA-seq reads (Table S9) and had homologues in *A. suum* (n = 13,727; 85.4%), *P. univalens* (n = 13,490; 83.9%), or *T. canis* (n = 13,873; 86.3%). A total of 11,135 (69.3%) genes were homologous among the four ascaridoid species, and 1283 (7.9%) were “unique” to *B. schroederi*, as no homologs were detected in any other ascaridoids for which genomic data were currently available (Figure S5). Using this gene set, we then performed functional annotation with public databases. In total, 14,968 (93.1%) and 14,831 (92.3%) genes had homologues in the Nr and InterPro databases, respectively. A total of 10,331 (64.3%) and 4414 (27.5%) genes contained Pfam domains and at least one transmembrane domain, respectively (Table S10). Notably, 3465 (21.6%) of the 16,072 *B. schroederi* genes had orthologs linked to one or more of 134 known Kyoto Encyclopedia of Genes and Genomes (KEGG) biological pathways. Most of these genes can also be mapped to those in *Caenorhabditis elegans* (Table S11). Moreover, 28 genes were assigned to four groups of antimicrobial effectors: cecropins, saposins, neuropeptide-like proteins (NLPs), and nematode antimicrobial peptides (AMPs) (Table S12; File S1).

### Host-shift events in ascaridoid lineages indicate accelerated host**–**parasite adaptations

**Figure 2:**
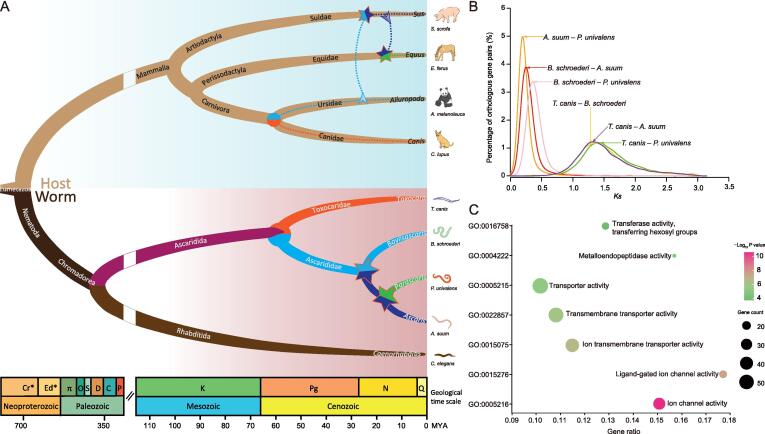
**Phylogeny and inferred host shifts of *B. schroederi* and related ascaridoid species** **A.** The phylogenetic tree and divergence time of four ascaridoids and their hosts. The upper branches (azure background) show the relationship of the ascaridoid hosts including pigs, horses, giant pandas and dogs, which is estimated based on previous studies [22], [24]. Lower branches (light pink background) show the relationship of the ascaridoids included in this study, inferred based on 329 single-copy orthologs using RAxML and BEAST2. The colors in the lower branches indicate different ascaridoid species. The circle indicates the species differentiation between *T. canis* and Ascaridida species (*i.e.*, *B. schroederi*, *A. suum*, and *P. univalens*), and the pentagrams indicate the host-shift events and speciation in the Ascaridida lineage. The dashed lines indicate that the ascaridoids were acquired via predation or food webs before settling in their own hosts. The arrows indicate the direction of host shifts. **B.***Ks* distribution of orthologous genes among the four ascaridoids. **C.** GO enrichment of differentiation genes, which are mainly enriched in transferase, transporter, metalloendopeptidase, transmembrane transporter, and ion channel. GO, Gene Ontology.

**Supplementary Figure S6:**
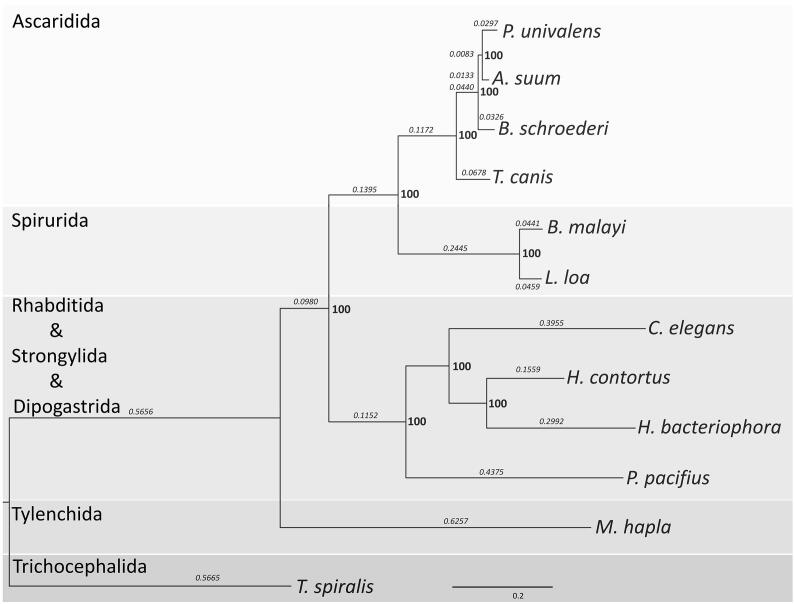


**Supplementary Figure S7:**
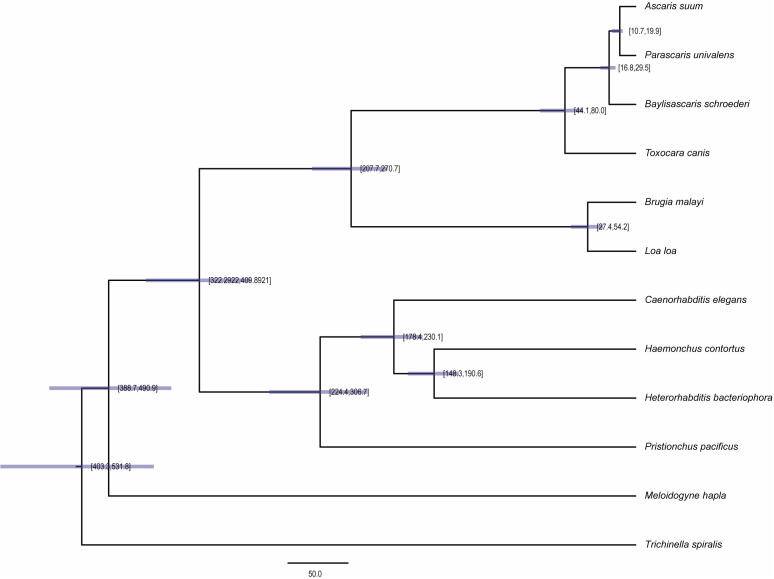


To determine the evolution of ascaridoid parasites in the context of nematodes, we inferred the phylogeny from 329 single-copy core orthologs across 12 nematode genomes using the maximum likelihood method (Figure 2A, Figure S6). Based on the phylogeny, the orders Ascaridida, Spirurina, and Rhabditina were each treated as a monophyletic group in the phylum Nematoda, in accordance with the previously proposed molecular phylogeny [10], [11]. Within Ascaridida, we found that the giant panda roundworm *B. schroederi* was more closely related to *A. suum* and *P. univalens* than to *T. canis*, supporting current taxonomic classifications of these species [12]. Combining the phylogeny and previously published divergence dates with the fossil record [13], [14], [15], [16], we estimated the divergence time of the ascaridoid parasites and other nematodes. The result showed that Ascaridida and Spirurina separated 238 million years ago (MYA), and Ascaridida and Spirurina diverged from Rhabditina about 365 MYA (Figure 2A, Figure S7). Furthermore, among the order Ascaridida, *Baylisascaris* separated from *Ascaris* and *Parascaris* about 22 MYA in the early Miocene, while *Baylisascaris* and *Toxocara* diverged about 59 MYA in the late Paleocene. Both of these divergence times appeared to postdate the splits respectively between *Baylisascaris* and *Ascaris*/*Parascaris* (∼ 70 MYA) and between *Baylisascaris* and *Toxocara* (∼ 200 MYA), which were previously estimated based on partial nuclear and mitochondrial genes [17], but agreed with an earlier speculation in which species of *Ascaris* and *Caenorhabditis* diverged at ∼ 400 MYA [18].

**Supplementary Figure S8:**
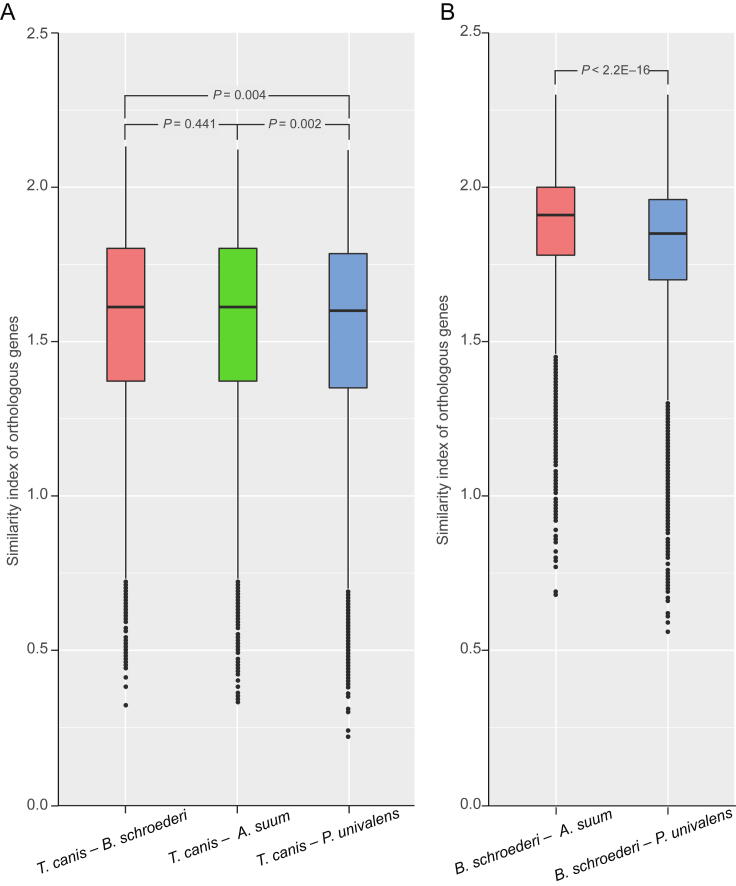


Moreover, an assumed ‘host shift’ theory was developed based on the following results from our analysis of the divergence time and topological relationships among the four ascaridoid species and their host animals. First, the divergence time (59 MYA) of *T. canis* and *B. schroederi* was close to that of their hosts dog and giant panda (61 MYA) [19], and the common ancestor of *B. schroederi*, *A. suum*, and *P. univalens* split from the ancestor of *T. canis* following the differentiation of their own hosts. Secondly, *P. univalens* shared a lower similarity with *T. canis* than with *B. schroederi* and *A. suum*. Next, the divergence of the three ascaridoid species postdated the differentiation of their own hosts, as the giant panda and horse diverged about 89.6 MYA [19], and the pig and horse diverged about 105.7 MYA [20]. Additionally, *A. suum* was more closely related to *P. univalens* than to *B. schroederi*. Finally, the similarity of orthologous genes between *B. schroederi* and *A. suum* was higher (Wilcoxon signed rank test, *P* < 2.2E−16) than that of orthologous genes between *B. schroederi* and *P. univalens* (Figure S8). We concluded that the ancestor of these three ascaridoids first colonized the panda ancestor; the pig ancestor then acquired the ascaridoid from panda ancestors via predation or food webs and hosted this parasitism until the formation of its own ascaridoid, *A. suum*. The horse ancestor then acquired the ascaridoid from pig ancestors and gave rise to the horse ascaridoid, *P. univalens* (Figure 2A). This ‘host shift’ hypothesis could be further supported by the synonymous substitution rate (*Ks*) distribution of orthologous genes between the four ascaridoid species. The peak of the *Ks* distribution of orthologous genes between *B. schroederi* and *A. suum* was significantly (*P* < 2.2E–16) skewed from that between *B. schroederi* and *P. univalens* (Figure 2B). This refuted the theoretical observation that *A. suum* and *P. univalens* grouped in one branch and either *A. suum* and *B. schroederi* or *P. univalens* and *B. schroederi* in the other should exhibit an overlapping peak in the *Ks* distribution. Furthermore, the peak value of the *Ks* distribution between *B. schroederi* and *A. suum* was lower than that found in the *Ks* distribution between *B. schroederi* and *P. univalens*.

In addition, based on pairwise comparisons of diverged genes retrieved from orthologous genes between *B. schroederi*, *A. suum*, and *P. univalens*, we found that some diverged genes were shared among these three ascaridoids (Table S13). Gene Ontology (GO) enrichment and functional annotations showed that most of the genes were enriched in ion channel activity (GO:0005216, *P* = 2.6E–05), transporter activity (GO:0005215, *P* = 3.5E–03), metalloendopeptidase activity (GO:0004222, *P* = 7.7E–03), and transferase activity/transferring hexosyl groups (GO:0016758, *P* = 2.4E–02) (Figure 2C; Table S14), which play roles as material transport-related carriers, including sugar (and other) transporters, transmembrane amino acid transporter proteins, ABC transporters, and ammonium transporter/ion transport proteins, and have functions in material metabolism, including glycolysis/gluconeogenesis, biosynthesis of amino acids, amino sugar and nucleotide sugar metabolism, glycerophospholipid metabolism, and purine/pyrimidine metabolism (Table S15). These findings indicated that the host shifts may have accelerated the divergence of orthologs among these three ascaridoids to allow better adaptations to their new nutritional environment due to differences in host feedings. Additionally, several genes likely involved in host–parasite immune interactions, including those encoding immunoglobulins, lectins, flavin-containing amine oxidoreductases, thioredoxins, serpins, and tetraspanins, were identified. For instance, flavin-containing amine oxidoreductases might modulate the levels of host amines (*e.g.*, histamine) and trigger tissue damage in nematode infections [21], [22]. Tetraspanins bind the Fc domain of immunoglobulin G (IgG) antibodies and might help the parasites evade host immune recognition and complement activation [23], [24].

### Strengthened gene functions in ***B. schroederi*** contribute to survival in a nutrient-limited environment

**Figure 3:**
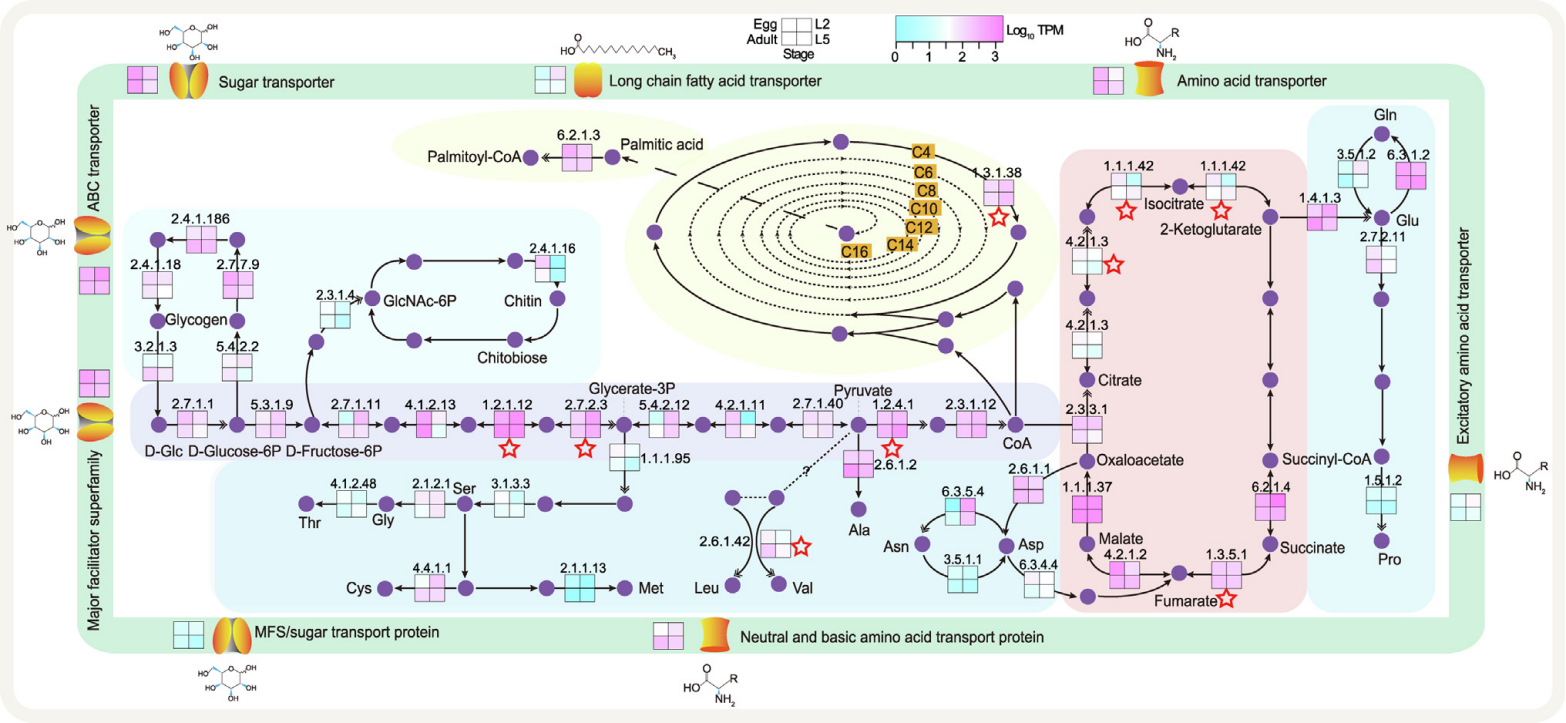
**Nutrient transport and metabolism of *B. schroederi*** *B. schroederi* absorbs nutrients such as sugars, amino acids, and fatty acids from the gut of giant pandas through transport proteins to enter cells and carry out metabolic processes in the cells. Different colored backgrounds indicate different metabolic pathways: green, sugar metabolism; peach, tricarboxylic acid cycle; blue, amino acid synthesis; beige, fatty acid synthesis; lightcyan, glycogen synthesis; and pink, chitin metabolism. The purple circles indicate metabolic substrates or products. The number indicates the Enzyme Commission number of the enzyme, the double arrow shows the expanded enzyme-coding gene that catalyzes the indicated step, and the red asterisk means the enzyme-coding gene that was under positive selection. The four colored boxes indicate the expression levels of the enzyme-coding genes during the four developmental stages including Egg, L2, L5, and Adult.

To survive in an environment where nutrients are relatively scarce and simple, *B. schroederi* has been thought to enhance functions of genes related to basal energy expenditure by positive selection and expansion to meet its own requirements for the metabolism of major nutrients. Comparative genomic analysis showed that genes encoding transporters were under expansion and/or positive selection in *B. schroederi*. The ABC transporter (Pfam: PF00005), sugar (and other) transporter (Pfam: PF00083), major facilitator superfamily (Pfam: PF07690), MFS/sugar transport protein (Pfam: PF13347), neutral and basic amino acid transport protein (solute carrier family 3; Pfam: PF16028), transmembrane amino acid transporter protein (Pfam: PF01490), excitatory amino acid transporter (sodium:dicarboxylate symporter family; Pfam: PF00375), and long-chain fatty acid transport protein (AMP-binding enzyme; Pfam: PF00501) gene families were expanded; and the first two were also identified to be under positive selection (Figure 3; Tables S16–S19). Moreover, genes encoding enzymes for glycolysis/gluconeogenesis and amino acid/fatty acid biosynthesis were found to be enriched in segmental and tandem gene duplications of the *B. schroederi* genome (Tables S20–S23). Combined, these results suggest an increasing ability to transport/metabolize sugars, amino acids, and fatty acids in *B. schroederi*.

In *B. schroederi* metabolism, increased sugar transport capacity enhanced sugar metabolism-related pathways to provide energy, and increased intermediate products improved the efficiency of nutrient conversion (*e.g.*, amino acids and fatty acids). For example, the glycolysis/gluconeogenesis and citrate cycle (TCA cycle), hexokinase (EC:2.7.1.1) pyruvate dehydrogenase E2 component (dihydrolipoamide acetyltransferase) (EC:2.3.1.12), citrate synthase (EC:2.3.3.1), and succinyl-CoA synthetase alpha subunit (EC:6.2.1.4) gene families were expanded (Figure 3; Tables S16 and S17). Moreover, the glyceraldehyde 3-phosphate dehydrogenase (EC:1.2.1.12), isocitrate dehydrogenase (EC:1.1.1.42), succinate dehydrogenase (ubiquinone) iron-sulfur subunit (EC:1.3.5.1), and related gene families were identified to be under positive selection (Tables S18 and S19). Interestingly, the phosphoglycerate kinase (EC:2.7.2.3), pyruvate dehydrogenase E1 component alpha subunit (EC:1.2.4.1), and aconitate hydratase (EC:4.2.1.3) genes were under both expansion and positive selection. These results showed that enhancing the function of enzymes related to glucose metabolism promotes the formation of intermediate products and the conversion of other nutrients.

In addition, important enzymes for amino acid and fatty acid biosynthesis were also expanded in *B. schroederi*. In the biosynthesis of amino acids, the glutamate dehydrogenase [NAD(P)+] (EC:1.4.1.3), D-3-phosphoglycerate dehydrogenase/2-oxoglutarate reductase (EC:1.1.1.95), pyrroline-5-carboxylate reductase (EC:1.5.1.2), branched-chain amino acid aminotransferase (EC:2.6.1.42), phosphoglycerate kinase (EC:2.7.2.3), aconitate hydratase (EC:4.2.1.3), and asparagine synthase (glutamine-hydrolyzing) (EC:6.3.5.4) gene families were expanded (Figure 3; Tables S16 and S17). The glyceraldehyde 3-phosphate dehydrogenase (EC:1.2.1.12) and branched-chain amino acid aminotransferase (EC:2.6.1.42) gene families were under positive selection (Tables S18 and S19). Interestingly, the branched-chain amino acid aminotransferase (EC:2.6.1.42) gene family was under both expansion and positive selection. Simultaneously, similar metabolic processes occurred in fatty acid biosynthesis. The long-chain acyl-CoA synthetase (EC:6.2.1.3) gene family was expanded, and the mitochondrial enoyl-(acyl-carrier protein) reductase/trans-2-enoyl-CoA reductase (EC:1.3.1.38) gene family was positively selected (Figure 3; Tables S18 and S19). All these metabolic processes indicated that *B. schroederi* could use host nutrients to synthesize nutrients to compensate for inadequate nutrition in the giant panda gut. The transcriptome analysis showed that these genes were highly expressed in the intestine of worms, which further provided strong evidence that the worms replenish nutrition by enhancing metabolic activities to adapt well to the nutritional environment of the giant panda gut.

### Thickened cuticle in ***B. schroederi*** confers protection against the fiber-enriched intestinal environment of giant pandas

**Figure 4:**
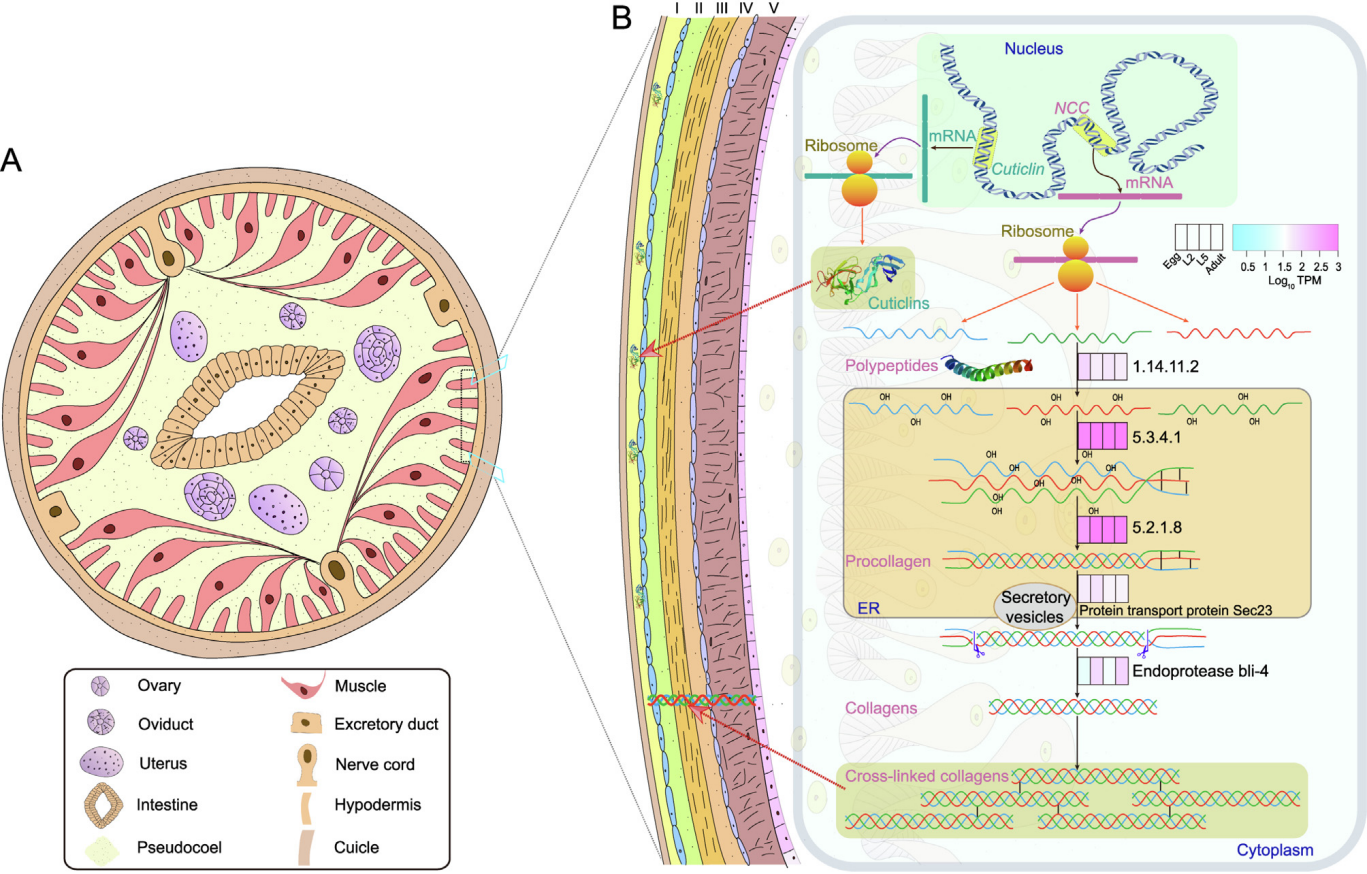
**Composition of related gene expression in the *B. schroederi* cuticle** **A.** Diagram model of the transverse section through the midbody of *B. schroederi*. **B.** Cuticle of *B. schroederi* is magnified from (A) to illustrate its structure and biosynthesis. The cuticle includes the surface coat (I), epicuticle (II), and the cortical (III), medial (VI), and basal layers (V), and its composition is mostly made up of collagens and cuticlins. Cuticlins are enzymatically polymerized to constrict the seam cell-derived cuticle and form the distinctive cuticular alae, which are predominant in the outermost cortical layers; while collagens are synthesized through a multistep process that includes prolyl 4-hydroxylation, procollagen registration and trimerization, transport from the endoplasmic reticulum, and procollagen processing and cross-linking, which results in construction of the major component of the extracellular matrix of the epicuticle and the cortical, medial, and basal layers of the nematode cuticle. Transcriptome analysis showed significantly (corrected *P* value ≤ 0.05; fold change ≥ 2) differential expression of the genes involved in the biosynthesis of cuticle collagens and cuticlins during the development of *B. schroederi*. For expression levels of genes related to biosynthesis of collagens, the four color boxes from left to right representing four stages (Egg, L2, L5, and Adult) were used to indicate expression levels, with pink and cyan indicating high and low deviation from the consensus profile, respectively.

The nematode cuticle plays a protective role against a variety of external biotic and abiotic stresses. It is composed of five layers and is formed from collagens, cuticlins, chitin, and small amounts of lipids [25], [26], [27]. Collagen is the structural protein in cuticles and comprises the major component of the extracellular matrix. This protein is synthesized through a multistep process that includes prolyl 4-hydroxylation, procollagen registration and trimerization, transport from the endoplasmic reticulum, and procollagen processing and cross-linking, similar to the phenomenon in vertebrates [26], [27], [28]. Cuticlin is another structural component of the cuticle. It is abundant in the outermost cortical layers and hypothesized to be enzymatically polymerized to constrict the seam cell-derived cuticle and thereby form the distinctive cuticular alae in *C. elegans* [25], [26], [29]. In this study, we identified 158 gene copies encoding cuticle collagens, and each expressed product contained a nematode cuticle collagen N-terminal domain and/or collagen triple helix repeats (n = 20) (Figure 4; Table S24). These genes showed significantly differential expression during the development of *B. schroederi* and presented quite high expression levels in the L5 and Adult stages, indicating a high demand for collagens in the formation of the worm cuticle. Collagen synthesis was catalyzed by prolyl 4-hydroxylase (EC:1.14.11.2), protein disulfide-isomerase (EC:5.3.4.1), and peptidyl-prolyl *cis-trans* isomerase A (EC:5.2.1.8). This step was followed by cleavage at the N- and C-termini by endoprotease and zinc metalloproteinase and then maturation and structural cross-linking by dual oxidase (EC:1.6.3.1). These enzymes, except for zinc metalloproteinase and dual oxidase, were highly expressed at the L5 and Adult stages, contributing to the formation of a thick exoskeleton for bodily protection against the bamboo-dominated intestinal environment of the giant panda. In addition, 32 cuticlins (zona pellucida-like domain; Pfam: PF00100) were also identified in *B. schroederi* (Figure 4; Table S24).

**Figure 5:**
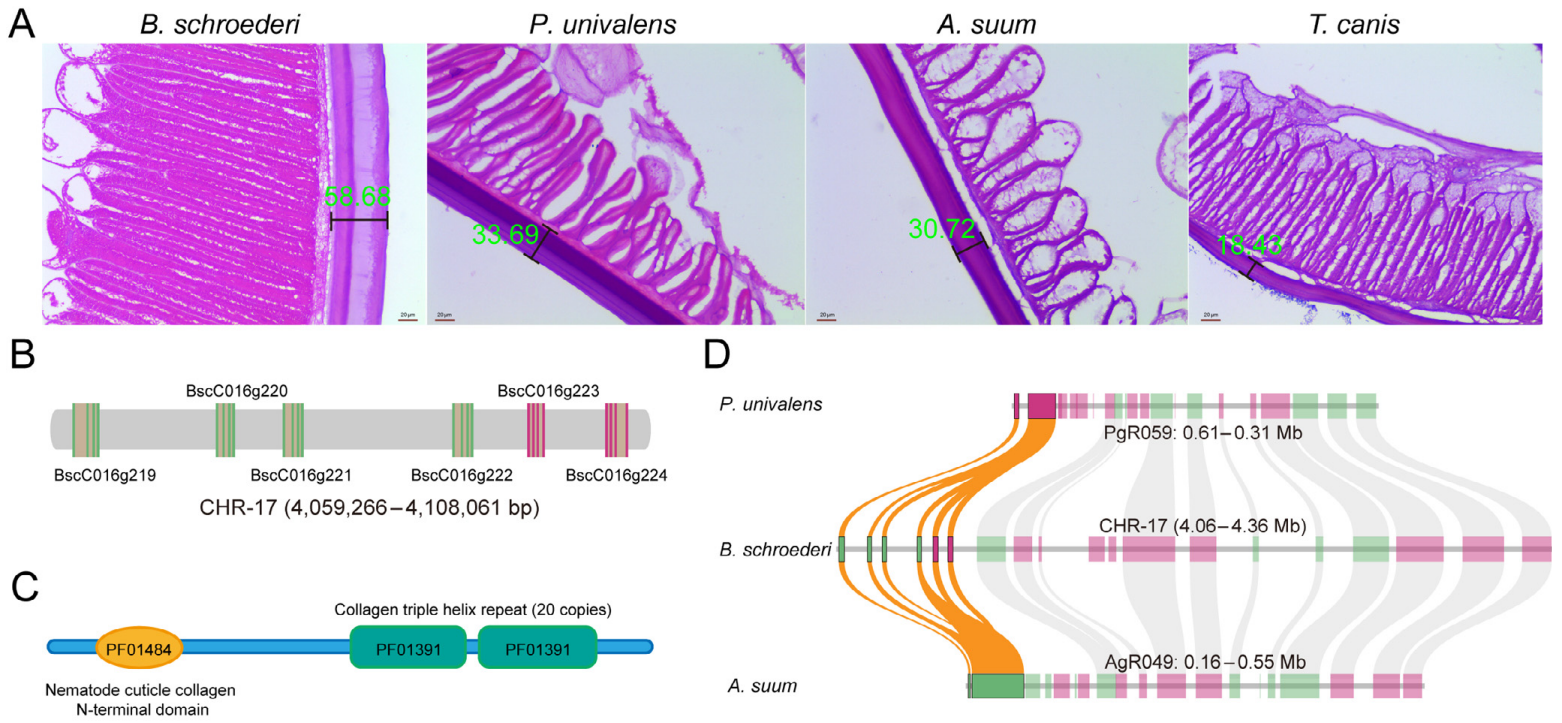
**Cuticle thickness and expanded genes related to cuticle biosynthesis in ascaridoids** **A.** Cuticle thickness of *B. schroederi*, *A. suum*, *P. univalens*, and *T. canis*. The scale bars denote 20 µm. **B.** The genes encoding cuticle collagens are presented in tandem on the genome. Green represents the genes on the positive strand while pink represents those on the negative strand. **C.** The Pfam domain of cuticle collagens that includes one “nematode cuticle collagen N-terminal domain” and two “collagen triple helix repeat (20 copies)”. **D.** Genes encoding nematode cuticle collagens were tandemly duplicated in syntenic blocks between *B. schroederi* and *A. suum*/*P. univalens*. The highlighted colors represent genes encoding nematode cuticle collagens, and the dimmed colors represent other genes in syntenic blocks.

**Supplementary Figure S9:**
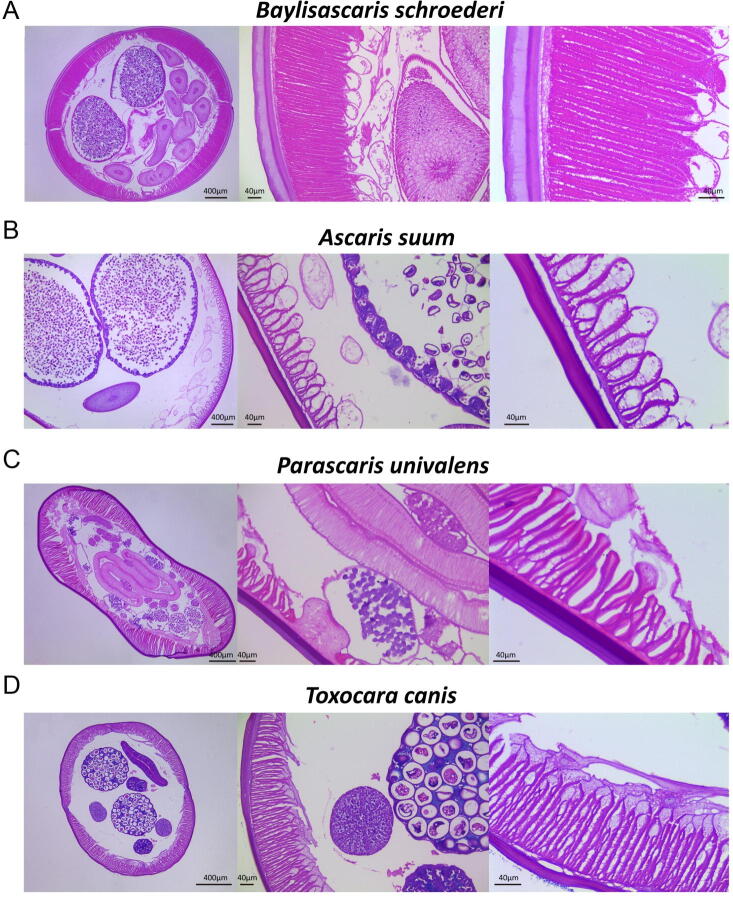


**Supplementary Figure S10:**
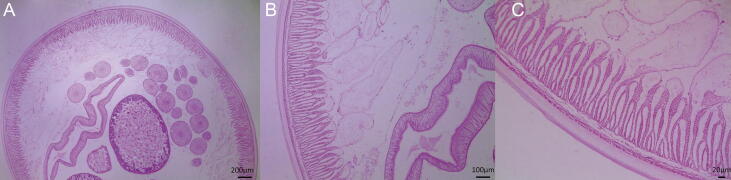


**Supplementary Figure S11:**
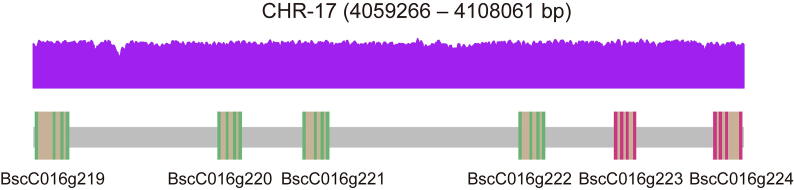


Interestingly, our speculation regarding thickened cuticles in *B. schroederi* was confirmed by histological examinations (Figure 5A, Figure S9; Table S25). It was clear that the *B. schroederi* cuticle was significantly thickened compared with the other three ascaridoids *A. suum*, *P. univalens*, and *T. canis* (*P* < 0.01). To determine whether this thickness difference was derived from species variations among ascaridoids, we also introduced the ursine *Baylisascaris transfuga* for congeneric comparisons (Figure S10). Notably, *B. schroederi* had a markedly thicker cuticle than *B. transfuga* (*P* < 0.01). We further compared the cuticle-related genes among *B. schroederi*, *A. suum*, *P. univalens*, and *T. canis* (*B. transfuga* was not included because its genome has not yet been sequenced), and found that collagen genes were expanded after the separation of *B. schroederi* and *Ascaris*/*Parascaris* (Figure 5B and C) and highly expressed at the L5 and Adult stages of *B. schroederi* (Tables S18 and S19). Structural analysis showed that most of these collagen genes were present in collinear regions among *B. schroederi*, *A. suum*, and *P. univalens*, in the form of tandem repeats with equal sequencing depths (Figure 5D, Figure S11), suggesting the authenticity of gene expansion events. In addition, genes encoding peptidyl-prolyl *cis*-*trans* isomerases (EC:5.2.1.8) and cuticle collagens were also positively selected. Such adaptive selections would undoubtedly enhance the functions of these genes in the cuticle of *B. schroederi*. Combined, these results suggested that through copy-number increases and the functional strengthening of genes involved in cuticle collagen formation, *B. schroederi* has evolved a thicker cuticle as armor to protect itself from the sharp-edged bamboo culm/branch-enriched intestinal environment during parasitism in the giant panda.

### Differentially expressed genes in the four life stages of ***B. schroederi***

**Figure 6:**
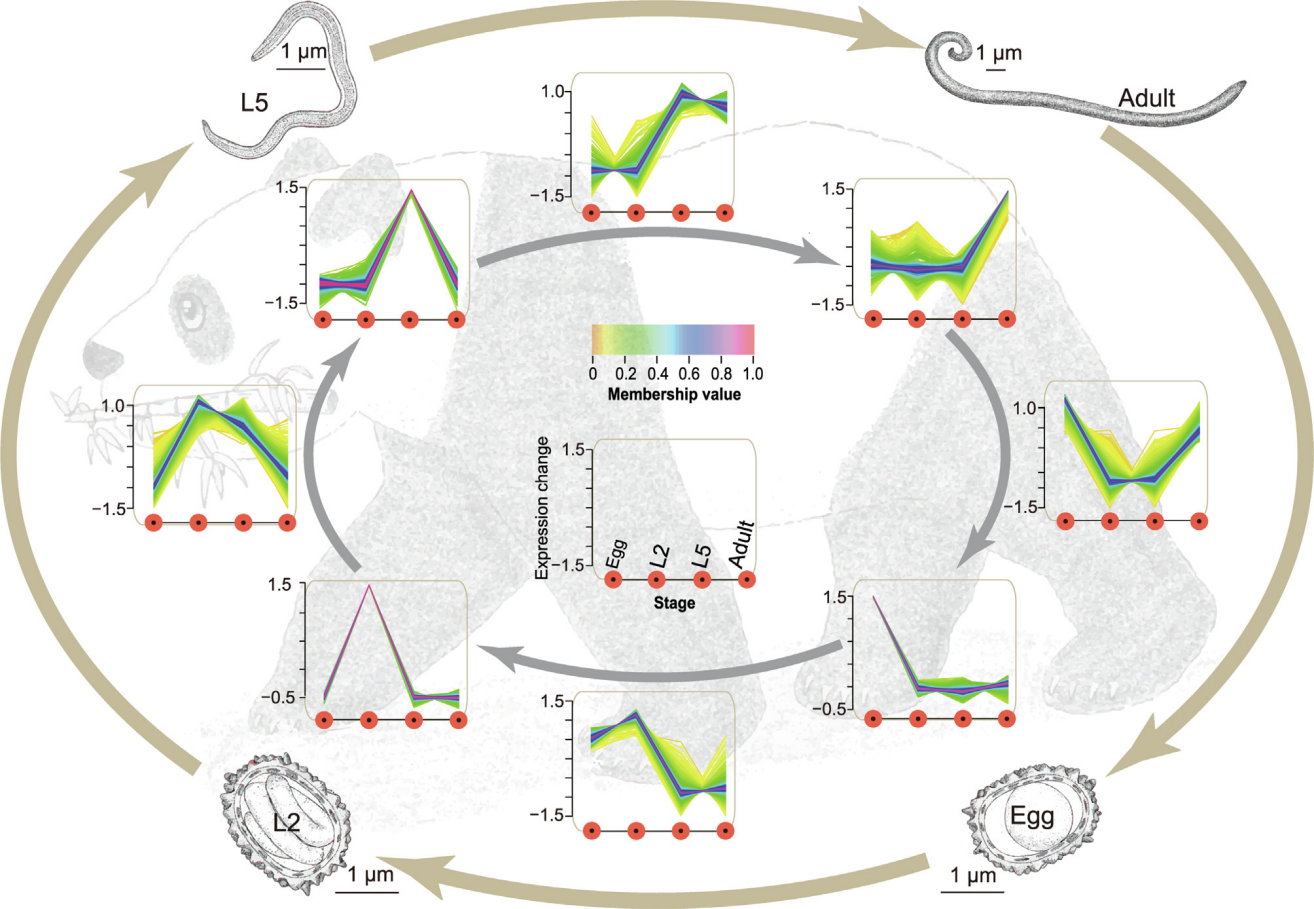
**Example of differentially expressed gene clusters during the *B. schroederi* life cycle** The developmental transcriptomes of *B. schroederi* were sequenced in triplicate at four stages across the lifecycle (1st order): Egg, L2, L5, and Adult. A subset of the 11,510 differentially expressed genes (corrected *P* value ≤ 0.001; fold change ≥ 4) were grouped into expression clusters describing the genes specifically up-regulated at various life stages. Clusters that uniquely describe each life stage and describe two life stages are identified (2nd order). For all expression clusters, the expression change (vertical axis) for each gene is plotted at each life stage (horizontal axis) in the following order: Egg, L2, L5, and Adult. The colors of the lines in each cluster show the membership values which were calculated by mfuzzy c-means algorithm [67], and the larger value represents a stronger correlation between a gene and corresponding expression cluster. The orange and red shades indicated low and high membership values, respectively.

**Supplementary Figure S12:**
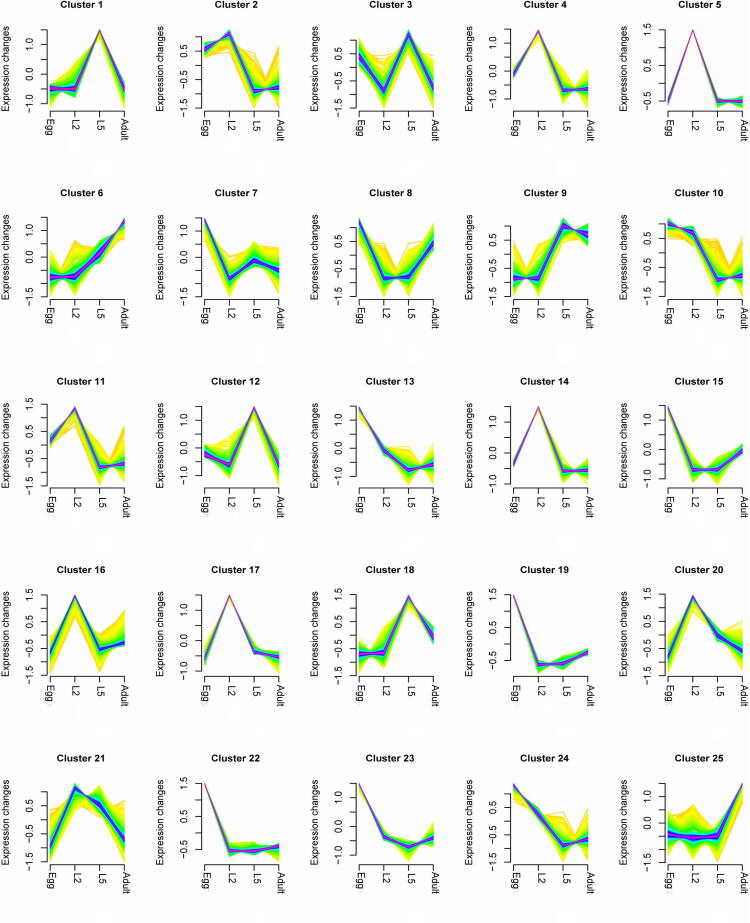


To understand the developmental process of *B. schroederi*, we profiled genes that were differentially expressed among the four developmental stages (Egg, L2, L5, and Adult) across the lifecycle (Figure 6; Tables S26 and S27). We found that 14,178 genes were significantly (corrected *P* value ≤ 0.05; fold change ≥ 2) expressed in at least one stage, and 11,510 genes were differentially expressed among the four life stages. We grouped these 11,510 genes into expression clusters to uniquely describe each life stage (Egg, L2, L5, or Adult) and two life stages (Egg and L2; L2 and L5; L5 and Adult; or Adult and Egg), and included expression clusters showing an increase or a decrease in expression corresponding to some transitions through the lifecycle (Figure S12). The genes with up-regulated expression during development from the Egg to L2 stage included those involved in the chromatin assembly, cellular component organization, and morphogenesis (Tables S26 and S27), in agreement with the progression from embryonation to the motile and infective larval stage. The L2 stage was characterized by an increased number of expressed genes related to signaling pathways, cell communication, response to stimulus, cellular homeostasis, and molecular binding and/or transport (Tables S26 and S27). This mirrored the larval adaptation to external conditions and the increased need to detoxify endogenous build-up wastes, consistent with the results from previous studies in *T. canis* [9], [30]. In addition, we observed a decrease in the transcription of genes associated with catalytic activity, oxidoreductase activity, electron transfer activity, organic cyclic compound metabolic process, amino acid metabolic process, and lipid metabolic process observed at the L2 stage (Table S27). This supported the notion that the larvae experience a quiescent state that allows their adaptation to a reduced metabolic rate in order to survive outside for extended periods [31], [32].

The transition from the tissue/organ migrating larval stage to the intestine-inhabiting L5 stage was reflected by the significantly up-regulated expression of genes involved in metabolic processes, oxygen transport, actin cytoskeleton, and cuticle development (Table S26). The expression of genes encoding protein kinases/kinases, peptidases, phosphatases, transferases, and hydrolases, which are possibly associated with food degradation and digestion in *A. suum* and *T. canis* [8], [9], were also up-regulated (Tables S26 and S27). In addition, we noted significantly increased expression of genes encoding enzymes related to cell redox homeostasis, including glutathione S-transferase, arylesterase, oxidoreductase, and glutathione peroxidase and/or peroxiredoxin. These observations likely reflect the maintenance of the redox balance in response to the accumulation of the end products from anaerobic metabolism and the clearance of reactive oxygen species from endogenous metabolic activities during infection. The development process from L5 to adulthood was characterized by gene sets enriched in genes associated with metabolic processes, hormone-mediated growth and development, and embryogenesis in adult females (Tables S26 and S27). For instance, the expression of genes involved in amino sugar/carbohydrate metabolism, insulin-like growth factor binding, steroid mediated growth, and embryonic division was significantly up-regulated, and this up-regulation was accompanied by increased expression of genes involved in DNA replication/repair during this transition.

### Identification of prioritized drug targets and vaccine candidates

Currently, the excessive use of a small number of drug classes for the treatment of *B. schroederi* infection has resulted in the emergence of drug resistance in the captive panda population [33], [34]. The *B. schroederi* genome provides an alternative approach to drug target discovery for new interventions [35]. We identified 1093 essential genes linked to lethal-gene-knockdown phenotypes in *C. elegans*, and 454 of these were shared with the ChEMBL database (Table S28). Further, 194 of these 454 genes were deemed one-copy orthologs absent in hosts (Table S28). We focused on this gene set and gave the highest priority to genes that were inferred to be highly expressed and to function as enzymatic chokepoints [32], [36]. Finally, we identified several druggable candidates, including threonine/serine peptidases (n = 2), metalloenzymes (n = 1), serine/threonine kinase (n = 1), and Ser/Thr phosphatases (n = 2) (Table S28), representing known targets of norcantharidin analogs with nematocidal activity [37], [38]. We also identified four transporters in the *B. schroederi* genome (Table S28). The transporters represent validated targets for many current antihelmintics, including imidazothiazole derivatives (including levamisole), macrocyclic lactones, cyclic depsipeptides, and amino-acetonitrile derivatives (AADs) [32], [39], [40]. These prioritized drug targets could prompt the rational design of anthelmintics, particularly when these targets exert antinematodal effects *in vitro*, as demonstrated through larval development assays, and *in vivo* in the giant panda (unpublished data).

In addition to drug target discovery, we also analyzed the *B. schroederi* genome for suitable vaccine targets based on genes predicted to encode the excretory/secretory (E/S) proteins that are secreted into the host, are exposed to the host's immune system, and can modulate the immune system of the host to promote parasitism. We focused on the predicted E/S proteins (Table S29; File S1), particularly those expressed during the parasitic stages of *B. schroederi* and representing parasite-specific genes (no orthologs in host mammals). Such screening yielded 85 proteins, with protease inhibitors predominating (n = 61). Conspicuous among them were thioredoxins that were highly expressed at the L5 and Adult stages (Table S30) and cysteine peptidases that have been under close scrutiny as vaccine candidates [41]. Moreover, other E/S proteins, including serine peptidases and their inhibitors as well as serpins, were also up-regulated within the parasitic stages; their feasibility for the development of vaccines remains under evaluation [42].

## Discussion

The diet of the giant panda is made up almost exclusively of low-protein, high-fiber bamboos [1]. This high degree of food specialization not only shapes the panda’s behaviors to respond to food challenges, but also promotes the adaptation of its tract-inhabiting parasitic nematodes to the harsh (sharp-edged bamboo culm/branch-enriched) intestinal environment of the pandas. Increased lines of evidence show that *B. schroederi* can grow to a body size comparable to that of *A. suum* in pigs and *P. univalens* in horses [3], [6], which suggests that this parasite has highly evolved from these other species to adapt to its host. To explore the speciation of *B. schroederi* and its parasitic adaptation to the panda, in this study, we decoded its genome and found molecular clues related to host shift to illustrate the speciation and molecular evidence of the cuticle thickness and thus explain gut adaptation.

The genome-wide phylogeny showed that *B. schroederi* was closer to *A. suum* and *P. univalens* than to *T. canis*, and *A. suum* shared the closest relationship with *P. univalens*. However, comparative genomics revealed that the similarity of orthologous genes between *B. schroederi* and *A. suum* was higher than that between *B. schroederi* and *P. univalens*, and *P. univalens* shared the lowest similarity to *T. canis*. Considering morphometric and distribution data of these four ascaridoids as well as the historical biogeography and phylogeny of their unique hosts [17], [19], two host-shifting events likely occurred after the divergence of the common ancestor of ascaridoids between dogs and pandas. Specifically, the occurrence of *A. suum* appeared consistent with speciation following a host colonization event from pandas to pigs, apparently from a carnivoran source in sympatry. The occurrence of *P. univalens* appeared consistent with speciation following a host colonization event from pigs to horses, apparently from predation or food webs. Such history of host colonization is compatible with the current tree topology and coincides with historical evidence of the spatiotemporal coappearance of the panda ancestor *Ailurarctos lufengensis* and the pig ancestor *Sus scrofa* in the late Miocene and Pliocene. It also supports a recent molecular theory of a wide host-shifting origin for ascaridoid nematodes [17]. Nevertheless, the timing and geographic source for these ascaridoids cannot be elucidated in detail based on the currently available data and the reduced and relictual distribution of giant pandas. Future parasitological inventory among a wider host range in a region of sympatry is necessary to demonstrate how each ascaridoid species developed its a narrow host range and may now be limited to its present host [43]. Moreover, the apparent genomic differences among these ascaridoids, coupled with their divergent biogeographic and ecological histories, also suggest that this system is a good model for exploring the complexities of diversification and faunal assembly in the evolution of the host range and the associations among ascaridoids (*e.g.*, refs. [44], [45])

The nematode cuticle is a flexible and resilient exoskeleton composed primarily of cross-linked collagens, cuticlins, chitin, and small amounts of lipids [26], [27]. In *B. schroederi*, we observed accelerated evolution of genes related to cuticle biosynthesis, including significantly higher expression of genes encoding nematode cuticle collagens, chitin synthase, DP-N-acetylglucosamine pyrophosphorylase, and peritrophin-A chitin-binding proteins at the Adult stage. We also observed a significant expansion of genes such as collagens and peritrophin-A chitin-binding proteins compared with those in *T. canis*, *A. suum*, and *P. univalens* [7], [8], [9]. Such overexpression and expansion of genes responsible for cuticle formation in *B. schroederi* suggest its parasitic adaptation to the panda gut, which is filled with highly fibrous and sharp-edged components of bamboos. This conclusion was further supported by our analysis of positive selection, in which all the aforementioned genes were also positively selected in *B. schroederi* compared with those in *A. suum*, *P. univalens*, and *T. canis*. This molecular evidence, together with morphological and anatomical observations of these ascaridoid species and a congeneric *B. transfuga* from bears (Figure 4), reinforces the assumption that *B. schroederi* evolved a thicker cuticle as an armor against its harsh external environment in pandas. Given that the panda retains the alimentary tract of bears despite its unique diet, further studies that include *B. transfuga* for genome comparison are needed to confirm whether the significantly thickened cuticle is only present in *B. schroederi*.

In this study, we presented a chromosome-level genome assembly of the giant panda roundworm *B. schroederi* and described an evolutionary history marked by host-shift events in ascaridoid parasite lineages after host separations. We also identified uniquely increased cuticle thickness and efficient utilization of host nutrients in *B. schroederi*, which guarantee successful parasitism in giant pandas. We found a broad range of key molecule classes involved in host–parasite interplay and immunoregulation, including E/S peptidases and protease inhibitors highly expressed in parasitic L5 and Adult stages. These molecules lack homologs in the host, and therefore could serve as ideal targets for new and urgently needed interventions (drugs, vaccines, and diagnostic methods) to control *B. schroederi* infection in pandas. A combined use of the present genomic and transcriptomic datasets, as well as animal experimentation, will be advantageous for intervention optimization. These genome resources not only enable the transition from ‘single-molecule’ research to global molecular discovery in *B. schroederi* but also contribute to the conservation of giant pandas.

## Materials and methods

### Samples and preparations

Adult *B. schroederi* worms were collected from naturally infected giant pandas at the Chengdu Research Base of Giant Panda Breeding, Chengdu (Sichuan, China). Embryonated eggs were obtained from 2 cm proximal to the uteri of the females. The L2 larvae were harvested using established *in vitro B. schroederi* egg culture protocols [5], [8]. Briefly, after filtering through a 100-µm nylon sieve filter, washing with phosphate-buffered saline, and centrifugating, the egg suspension obtained from the uteri was placed into 100-mm culture dishes and maintained at ambient room temperature for at least 60 days to embryonate the eggs to an infective L2 stage. The eggs with well-formed and ensheathed larvae in the suspension were counted, and the suspension was then stored at 4 °C until use. L5 larvae (n = 25) were isolated from naturally infected captive giant pandas at the Chengdu Research Base of Giant Panda Breeding. These larvae, together with adult worms, were washed extensively in sterile physiological saline (37 °C), snap-frozen in liquid nitrogen, and then stored at −80 °C until use. All samples of other ascaridoid specimens, including *B. transfuga*, *A. suum*, *P. univalens*, and *T. canis*, were derived from naturally infected polar bears, pigs, horses, and dogs, respectively, and provided by the Department of Parasitology, College of Veterinary Medicine, Sichuan Agricultural University, China.

Genomic DNA was extracted from the freshly collected middle portion of *B. schroederi* to construct one paired-end library and one SMRT library using the Qiagen Genomic-tip 20/G kit (Catalog No. 10223, Qiagen, Düsseldorf, Germany) according to the manufacturer’s protocol. Briefly, a worm sample was mixed with 2 ml Buffer G2 containing 200 µg/ml RNase A and homogenized with a multi-bead shocker (Yasui-Kikai, Osaka, Japan). After the addition of 50 µl proteinase K (20 mg/ml; Catalog No. P6556, Sigma, St. Louis, MO), the lysate was incubated at 50 °C for overnight and centrifuged at 5000 *g* for 5 min at 4 °C. The aqueous phase was loaded onto a pre-equilibrated Qiagen Genomic-tip 20/G by gravity flow. DNA was eluted in Buffer QF, precipitated by isopropanol, and then desalted, concentrated, and resuspended in 10 mM Tris-HCl (pH 8.5). The quality and quantity of DNA were verified using agarose-gel electrophoresis, a Nanodrop micro-spectrophotometer (ThermoFisher Scientific, Waltham, MA), and a Qubit 3.0 Fluorometer (ThermoFisher Scientific, Carlsbad, CA).

Messenger RNAs (mRNAs) were isolated from *B. schroederi* embryonated eggs, L2 larvae, L5 larvae, and adult females, respectively, using TriPure isolation reagent (Catalog No. 11667165001, Roche, Mannheim, Germany). The flash-frozen samples for each stage were mixed with 2 ml Tripure reagent (Roche) and homogenized with a multi-bead shocker (Yasui-Kikai). Phase separation was performed by the addition of 0.4 ml chloroform and centrifugation at 12,000 *g* for 20 min at 4 °C, and the upper aqueous phase containing RNA was purified with a RNeasy Plus Mini Kit (Catalog No. 74134, Qiagen, Valencia, CA). RNA quality and quantity were evaluated by agarose-gel electrophoresis and Nanodrop micro-spectrophotometry (ThermoFisher Scientific). For paired-end RNA-seq, mRNA was purified from 10 mg of total RNA using the magnetic oligo-dT bead binding method and then fragmented, reverse-transcribed, end-repaired, and adaptor-ligated according to the manufacturer’s protocol (Illumina). Ligated products (300 bp) were excised through agarose-gel electrophoresis and enriched by PCR to construct cDNA libraries, which were sequenced on the Illumina HiSeq X Ten platform.

### Genome survey analysis

To survey the characterization of the *B. schroederi* genome, a 300-bp paired-end library was constructed, and a total of ∼ 10-Gb next-generation sequencing data were generated using the Illumina sequencing platform (Hiseq 4000) (Table S1). Adaptor sequences, PCR duplicates, and low-quality sequences were removed from the raw data to generate high-quality sequences. *K*-mer (17) statistics of the high-quality sequences were calculated by Jellyfish (version 2.1.3) [46] with “-C -m 17”. GenomeScope (version 2.0; http://qb.cshl.edu/genomescope/) software was used to estimate the size, heterozygosity rate (0.77%), and repeat content of the *B. schroederi* genome (Figure S13).

### Genome sequencing and assembly

One cell run of single-molecule long reads was generated with the PacBio Sequel II platform (Table S1). A total of 189-Gb long subreads (97,400,959,204 bp, ∼ 332× based on the estimated genome size) were generated and assembled *de novo* using CANU (version 1.8) [47]. The parameters were optimized for heterozygotic genomes according to the authors’ documentation. The initial CANU assembly was corrected using a combination of long and short reads using Pilon (version 1.23) [48] with the default parameters. Duplicated assembled haploid contigs were purged using Purge Haplotigs (version 1.1.1) [49], which reduced the assembly from 559 Mb to 299 Mb. A Hi-C library was constructed with *Hind*III as the digestion enzyme and sequenced in two batches with the Illumina HiSeq4000 and NovaSeq platforms. The purged contigs were anchored into superscaffolds using the Juicer and 3D-DNA pipelines. The generated assembly files were visualized and manually optimized using the built-in assembly tool (JBAT) of Juicebox [50]. Twenty-one pseudomolecules were preliminarily generated with the 3D-DNA pipeline. After breaking weak or ambiguous contact links between large TAD blocks and rebuilding the boundaries, a total of 27 pseudomolecules were generated. Note that these pseudomolecules did not represent complete chromosomes, and downstream synteny analysis with the *A. suum* genome showed that at least six molecules were likely partial chromosomes, which would reduce the number from 27 to 24. However, due to the lack of direct evidence of the karyotype of *B. schroederi*, we did not modify the result. Finally, the genome assembly contained 27 chromosome-level pseudomolecules and 123 unplaced scaffolds. The completeness of the assembly was assessed through BUSCO analysis (version 3.0.2, lineage dataset: nematoda_odb9; https://busco.ezlab.org/) and using RNA-seq data.

### Identification of repeat elements and non-coding RNAs

RepeatMasker (version 4.0.5; http://www.repeatmasker.org/) with the default parameters was applied to identify the dispersed repeats and tandem repeats. A species-specific repeat library was constructed with RepeatModeler (version 1.0.5, http://www.repeatmasker.org/). Using this library, repetitive sequences were further annotated and classified with RepeatMasker (http://www.repeatmasker.org/). The tRNA genes were predicted by tRNAscan-SE (version 1.3.1) [51] with general eukaryote parameters. The programs RNAmmer (version 1.2; http://www.cbs.dtu.dk/services/RNAmmer/) and rfam_scan.pl (version 1.2) were used to predict the large ribosomal subunit (LSU) and small ribosomal subunit (SSU) rRNA genes, respectively.

### Gene annotation

Protein-coding genes were annotated using a combination of *ab initio* gene prediction, homology-based gene prediction, and transcriptome-based prediction. A total of 12 RNA-seq libraries were used to construct the transcripts by applying the HISAT2 (version 2.1.0) and StringTie (version 1.3.4) pipelines [52]. All constructed transcripts were combined using TACO (version 0.7.3) [53]. The open reading frames (ORFs) on the transcripts were extracted with TransDecoder (version 5.5.0) [54]. The complete coding sequences (CDS) from the TransDecoder result were used as the training set for *ab initio* prediction, which was performed with the BRAKER2 pipeline (version 2.1.5) [55]. All protein sequences of the previously sequenced nematode genomes were mapped to the genome using GenomeThreader (version 1.7.1) [56]. EVidenceModeler (version 1.1.1) [57] was employed to integrate the results from the three prediction methods and thus generate a consensus gene set, and the resulting set was further curated by removing frameshift and redundancy using the GffRead (version 0.11.6; http://ccb.jhu.edu/software/stringtie/gff.shtml) tool from Cufflinks. Gene function annotation was performed using BLASTP (-evalue 1E–3) with public databases such as the nonredundant protein database (Nr) and the KEGG database (https://www.genome.jp/kegg/). InterproScan [58] was used to identify domains of the predicted proteins, assign GO terms to the predicted genes, and classify the functional annotations.

### Gene family analysis

Protein sequences from *B. schroederi* and 11 other nematodes (*A. suum*, *P. univalens*, *T. canis*, *Loa loa*, *Brugia malayi*, *Heterorhabditis bacteriophora*, *Haemonchus contortus*, *Pristionchus pacificus*, *C. elegans, Meloidogyne hapla*, and *Trichinella spiralis* retrieved from WormBase ParaSite, available at ftp://ftp.ebi.ac.uk/pub/databases/wormbase/parasite/releases/WBPS14/species/, under accession Nos. PRJNA62057, PRJNA386823, PRJNA248777, PRJNA37757, PRJNA10729, PRJNA13977, PRJEB506, PRJNA12644, PRJNA13758, PRJNA29083, and PRJNA12603, respectively) were used to analyze the gene family. Proteins with a length shorter than 30 amino acid (aa) or a frame shift were removed from the protein set, and the program OrthoFinder (version 2.3.3) [59] with the default parameters was used to construct the gene families and infer orthologous and paralogous genes. CAFE (version 3.0) [60] was utilized to identify the gene families that underwent expansion or contraction using the ultrametric tree created by BEAST2 and the estimated birth–death parameter λ.

### Homolog comparison

An all-to-all BLASTP (-evalue 1E–3 -outfmt 6) analysis of the proteins was performed to calculate the pairwise similarities, and the result was further filtered with “Identity > 30% and Query coverage ≥ 50%”. The *Ks* values of orthologous genes were calculated using codeml with the setting “runmodel = −2, CodonFreq = 2, model = 0, NSsites = 0”. To compare the similarities among Ascarididae species at the gene level, a similarity analysis of orthologous genes was performed with the following steps. First, *A. suum* and *P. univalens* were selected as the query species to analyze which species was more similar to the target species *B. schroederi*. Then, *B. schroederi*, *A. suum*, and *P. univalens* were selected as the query species to analyze which species was more similar to the target species *T. canis*. Next, the best-match orthologous genes of the target and query species were obtained from MCScan blocks, and the similarity index (SI) was calculated using the formula SI = S/L, where S represents the alignment score of a pair of proteins and L represents the target protein length. Finally, based on the SI value, the Wilcoxon signed-rank test was used to analyze whether the similarity between different query species and target species was significantly different (*P* ≤ 0.05, one-sided).

### Phylogeny construction

A total of 329 single-copy gene families were obtained, and the corresponding protein sequences were extracted. Individual protein alignment for each gene family was performed using Clustal Omega (version 1.2.1; http://www.clustal.org/omega/) with the default settings, and gaps in the alignments were removed using the program trimAl (version 1.4) [61] with the parameter “-nogap”. The alignments with a length of at least 100 aa were concatenated with a Perl script. The best amino acid substitution model for the protein alignment was estimated by ProtTest (version 3.4.2) [62] with the parameter “-IG -F -AIC -BIC -S 2 -all-distributions -tc 0.5”. The maximum likelihood tree was constructed using RAxML (version 8.0.24) [63] with the following parameters: 1) bootstrapping replicates, 200; 2) substitution model, LG + I + G + F; and 3) outgroup, *T. spiralis*.

### Divergence time estimation

The divergence time was estimated from the protein alignment by BEAST2 (version 2.5) [64] with the following parameters: 1) site Model, WAG + I + G + F; 2) clock model, relaxed clock log normal; 3) priors, calibrated Yule model; 4) time calibration; and 5) chain length per MCMC run, 10,000,000. Time calibration was defined as the split time (382–532 MYA) of Chromadorea and Enoplea [13], the split time (280–430 MYA) of *Pristionchus* and *Caenorhabditis* [14], and two fossil times (∼ 396 MYA and ∼ 240 MYA) for Enoplia [15] and Ascaridoidea [16], respectively. A consistent tree with divergence time was inferred by TreeAnnotator using a maximum clade credibility method and displayed using FigTree (https://github.com/rambaut/figtree/releases).

### Identification of positively selected genes

Orthologous genes of the four Ascarididae species (*B. schroederi*, *A. suum*, *P. univalens*, and *T. canis*) were extracted from the OrthoFinder results to identify positively selected genes (PSGs). Multiple protein sequence alignments were performed with Clustal Omega and converted to corresponding CDS alignments using an in-house Perl script. Gaps in the CDS alignments were removed with the program trimAl. The CodeML program with modified branch-site model A (model = 2, NSsites = 2), conducted in the PAML package (version 4.9) [65], was used to identify PSGs. The alternative hypothesis with estimated ω2 (fix_omega = 0 and initial omega = 1.5) and the corresponding null model with fixed ω2 = 1 (fix_omega = 1 and omega = 1) for the lineage *B. schroederi* (foreground branch) were used to calculate the omega values and log likelihood values, respectively. The likelihood ratio test for selection of the lineage of *B. schroederi* was performed based on the likelihood values obtained from the two models. Genes with *P* ≤ 0.05 were considered PSGs.

### Transcriptome analysis

Adaptor sequences, contaminants, and low-quality sequences were removed from the raw RNA-seq data. RSEM (version 1.1.17) [66] with the default parameters was used to map the high-quality reads to the transcripts and calculate the expression levels [transcripts per million (TPM) and read counts] of the protein-coding genes. Three replicates of each stage were used to reduce sampling bias. Differentially expressed genes among different developmental stages were detected with “edgeR” (version 3.30.3) from the R package (version 3.6.1) using the read counts of the genes. The genes with the false discovery rate-corrected *P* value ≤ 0.05 and fold change ≥ 2 were considered as differentially expressed genes. Clustering of the gene expression time-course data from the four stages of *B. schroederi* was performed with “Mfuzz” (version 2.48.0) [67].

### GO enrichment analysis

The tool BiNGO (version 3.0.4) implemented in Cytoscape (version 3.7.1; https://cytoscape.org/) software was used to analyze the GO enrichment of the genes from expanded gene families or differentially expressed genes with a hypergeometric test. The GO annotation profile of *B. schroederi* was constructed with an in-house Perl script, and the ontology file was obtained from the Gene Ontology web (http://geneontology.org/). GO terms with *P* ≤ 0.05 calculated by hypergeometric test were extracted for functional analysis.

### Histological processing and analysis

The ascaridoid adults were fixed in formalin and routinely processed for histology as described elsewhere [5]. Briefly, fresh adults of *B. schroederi* (n = 6), *A. suum* (n = 8), *P. univalens* (n = 8), and *T. canis* (n = 8), as well as ursine *B. transfuga* (n = 6), were fixed in 10% neutral phosphate-buffered formalin for 24 h. A portion (∼ 1.5 cm) of the middle body of each fixed worm was then cut, oriented transversally, and inserted into biopsy cassettes. All samples were washed in tap water and dehydrated by serial dilutions of alcohol (methyl, ethyl, and absolute ethyl). Paraffin embedding was performed with a Leica Tissue Processing station using a 12-h protocol. Paraffin blocks were prepared using a Leica inclusion station. Afterward, each block was cut into 3-μm tissue sections using a Leica microtome (Leica Instruments GmbH, Hubloch, Germany). Each slide was stained with hematoxylin and eosin (HE). For each worm, three slides were examined under an inverted light microscope (Olympus FSX100, Olympus Corporation, Japan), and the cuticle thickness of these five ascaridoid species were measured and compared. The data were expressed as the mean ± standard deviation (SD). Comparisons between ascaridoid species were performed by one-way analysis of variance (ANOVA), least significant difference (LSD), and Scheffe’s test using SPSS (IBM SPSS Statistics for Windows, version 20; Armonk, NY: IBM Corp.). *P* < 0.05 was considered to be significant.

### Identification of potential drug targets and vaccine candidates

All *B. schroederi* proteins were searched against lethal genes (WormBase WS226: WBPhenotype:0000050, WBPhenotype:0000054, WBPhenotype:0000060, WBPhenotype:0000062, and subphenotypes), the ChEMBL database (https://www.ebi.ac.uk/chembl/), and host proteins (http://panda.genomics.org.cn/download.jsp) using BLASTP (*E* ≤ 1 × 10^−10^). Genes with alignment length ratios and similarities higher than 0.5 were selected. Genes homologous to the lethal gene and ChEMBL database were then selected, genes homologous to the host were removed, and single-copy genes were further screened. The following equations were used to assign a score to each potential drug target gene:

  S_target_ = (S_l_ + S_c_) × 2 + S_t_ + S_e_
  (10) S_t_ = log(max(T_L5_, T_adult_))/log

S_l_ and S_c_ represent the SI values of genes homologous to the lethal gene and ChEMBL database, respectively, and T_L5_ and T_adult_ represent the gene expression (TPM) at the L5 and Adult stages, respectively. S_e_ equals 1 if the target gene encodes a protease, protein kinase, protein phosphorylase, transporter, or ion channel; otherwise, S_e_ is 0.

All proteins with signal peptides and one transmembrane structure domain were identified as E/S proteins. Proteases and protease inhibitors without host homology and TPM ≥ 1 were screened from secretory proteins as vaccine candidates.

## Ethical statement

This study was approved by the Animal Ethics Committee of Sichuan Agricultural University, China (Approval No. SYXK 2014-187) and the Wildlife Management and Animal Welfare Committee of China, and all procedures involving animals in the present study were in strict accordance with the Guide for the Care and Use of Laboratory Animals (National Research Council, Bethesda, MD, USA) and the recommendations in the ARRIVE guidelines (https://www.nc3rs.org.uk/arrive-guidelines).

## Data availability

All raw sequencing data (including the genome, transcriptome, and Hi-C data) described in this study have been deposited in the Genome Sequence Archive [68] at the National Genomics Data Center, Beijing Institute of Genomics, Chinese Academy of Sciences / China National Center for Bioinformation (GSA: CRA004125), which are publicly accessible at https://ngdc.cncb.ac.cn/gsa. The assembled genome and gene structures have also been deposited in the Genome Warehouse [69] at the National Genomics Data Center, Beijing Institute of Genomics, Chinese Academy of Sciences / China National Center for Bioinformation (GWH: GWHBAVG00000000), which are publicly accessible at https://ngdc.cncb.ac.cn/gwh.

## CRediT author statement

**Yue Xie:** Investigation, Formal analysis, Funding acquisition, Writing - original draft, Writing - review & editing. **Sen Wang:** Methodology, Formal analysis, Data curation, Visualization, Software, Writing - original draft. **Shuangyang Wu:** Methodology, Formal analysis, Visualization, Writing - original draft. **Shenghan Gao:** Methodology, Formal analysis, Funding acquisition, Writing - review & editing. **Qingshu Meng:** Formal analysis, Writing - review & editing. **Chengdong Wang:** Resources. **Jingchao Lan:** Formal analysis. **Li Luo:** Resources. **Xuan Zhou:** Investigation. **Jing Xu:** Formal analysis. **Xiaobin Gu:** Resources. **Ran He:** Investigation. **Zijiang Yang:** Formal analysis. **Xuerong Peng:** Resources. **Songnian Hu:** Conceptualization, Supervision, Resources, Writing - review & editing. **Guangyou Yang:** Conceptualization, Supervision, Resources, Funding acquisition, Writing - review & editing. All authors have read and approved the final manuscript.

## Competing interests

The authors have declared no competing interests.

## Figures and Tables

**Supplementary Figure S13:**