# Supplementary material for: Genome of the Giant Panda Roundworm Illuminates Its Host Shift and Parasitic Adaptation
Source: Genomics Proteomics Bioinformatics. 2021 Sep 3;20(2):366–81. doi: 10.1016/j.gpb.2021.08.002 (PMC9684166; doi:10.1016/j.gpb.2021.08.002)
Supplement: Supplementary File S1 [file mmc1.docx]

**File S1 Supplementary results**

**tRNA gene annotation**

A total of 6,190 transfer RNA (tRNA) genes were identified in the *B. schroederi* genome, and the copy numbers reflected their codon usages in protein-encoding regions (Figure S4, Tables S6 and S7). tRNA gene numbers often show wide diversity among species, even closely related species [1,2]. The number of tRNAs in *B. schroederi* was significantly higher than those in *T. canis* (2715), *P. univalens* (1179), *A. suum* (1152), and *C. elegans* (616). Further structural analysis showed that these tRNA genes appear in tandem in the *B. schroederi* genome (Table S7). In addition, the tRNA gene content in *B. schroederi* was consistent with the codon usage, and its correlation was higher than that found in *C. elegans* (Figure S4). Previous studies have shown that mismatches of codon usages of tRNAs can affect translation efficiency via multiple mechanisms, such as stress response programs [3,4]; however, the reason responsible for the expansion of tRNA genes in *B. schroederi* remains unclear, and further research is needed.

**ORFans**

Among *B. schroederi* gene set, 1104 genes had no homologs in the NR database and were classified as ORFans. To explore the possible origins and functions of these ORFans, their gene structures and expression levels across the *B. schroederi* lifecycle were analyzed (Figures S14 and S15). It was clear that all the ORFans contained a higher GC content, a longer intron, and a shorter exon than those of genes that had homologs in the public databases. In addition, only one exon was found in most ORFans, making their average numbers of exons much fewer than other annotated genes. A transcriptome analysis showed that among 1104 ORFans, 624 presented high expression levels at the different developmental stages of *B. schroederi*, including 275 in eggs, 393 in L2s, 384 in L5s, and 315 in adults (Figure S15). A further bioinformatics analysis combined with qRT-PCR validation is needed for expression confirmation of these ORFans in future.

**Nematode antimicrobial peptides**

Antimicrobial effectors are released by the innate immune system to defend against potential pathogens during the nematode lifecycle [5]. Four groups of antimicrobial effectors, including cecropins, saposins, NLPs, and nematode AMPs, were identified in *B. schroederi* (Table S12). Cecropins shows antibacterial and weak antifungal activity. According to previous studies, cecropins have only been identified in *A. suum* and are not found in *C. elegans*. Four cecropins were found in *B. schroederi* and exhibited quite high expression levels in adults, which likely suggests that these molecules are ascarid-specific antimicrobial effectors [5]. Saposins play a pivotal role in host-pathogen interactions and can be used as targets for vaccination [6]. Our results showed that five saposins showed stable expression at all stages (Table S12). NLPs were thought to function in resistance to fungal infection [7], and six NLPs were identified in *B. schroederi* and showed high expression at the L2 stage. The L2-stage expression pattern might indicate the most important antimicrobial effector of *B. schroederi* in this period. Nematode AMPs constitute the first line of defense against pathogens and can act directly on the pathogen membrane [8]. Four AMPs were identified, and the L5 and adult stages showed a high expression level pattern (Table S12). All of these antimicrobial effectors could be key immune modulators involved in adaptation to the complex environment of the gut.

**Expansion times of specialized nutrition-related genes in *B. schroederi***

To probe the possible time of nutrition specialization of *B. schroederi* in the coevolutionary history with the host giant panda, based on the divergence time of *B. schroederi* from *A. suum*/*P. univalens* that was inferred from their single-copy core orthologs, we estimated the expansion time of each metabolism-related gene using following the pipeline: (1) constructed the gene family tree using each metabolism-related gene from *B. schroederi*, *A. suum*, *P. univalens*, and *T. canis*; (2) gained sub-group from each gene family base on the tree topology, in which the genes of *B. schroederi* expanded after speciation; and finally (3) estimated expansion times of *B. schroederi* paralogs of metabolism-related genes using the split time of these four ascaridoids. We found that the expansion times of most targeted genes, including those encoding nutrition transport-related ABC transporter, sugar (and other) transporter, MFS/sugar transport protein, neutral and basic amino acid transport protein, transmembrane amino acid transporter protein, and long-chain fatty acid transport protein as well as nutrition metabolism-related hexokinase (EC:2.7.1.1), dihydrolipoate acetyltransferase (EC:2.3.1.12), putative citrate synthase (EC:2.3.3.1), dehydrogenase E1 (EC:1.2.4.1), glutamate dehydrogenase (EC:1.4.1.3), and asparagine synthetase (EC:6.3.5.4) (Figures S16 and S17), overlapped the time of the ancient giant panda diet shift that was inferred from isotope and molecular evidences [9–11]. These results, to a certain extent, supported the expansions of the metabolism-related genes of *B. schroederi*, which might correlate with its host diet changes. In addition, although it is impossible to estimate the time of positively selected genes using the current data, we observed that some positively selected genes also belonged to the expanded gene set, which implied that the times of metabolism-related gene genes that were under positive selection may be consistent with that of the giant panda diet shift as well.

**Cytoskeleton-related gene expansions**

To improve survival in giant pandas, *B. schroederi* enhances its cytoskeleton strength and adhesion to resist external pressure from the sharp-edged bamboo culm/branch-enriched intestinal environment of giant pandas. Specifically, the main protein gene families of the cytoskeleton were expanded (Table S24), and these included the tubulin, myosin, intermediate filament, troponin, merlin, paramyosin, and dynein families. The former five families, as well as vinculin and lamin, were also under positive selection (Tables S18 and S19). The enhancement of the cell adhesion ability also enhances related gene families, such as the cadherin family, and this gene family and the vinculin family were also under positive selection (Tables S18 and S19). In addition, the chondroitin proteoglycan gene family of chitin metabolic processes was expanded and under positive selection (Tables S18–S21). All of these results showed that *B. schroederi* could enhance the cell wall and cytoskeleton process to resist external pressure and thus adapt to the living environment of the giant panda gut.

**Key proteins associated with host-parasite interactions**

Parasite E/S proteins are known to play crucial roles in host-parasite interactions and host immunomodulation and evasion [12–17]. Based on the current understanding of these aspects, we compiled the E/S proteins of *B. schroederi*, particularly secreted peptidases that are able to induce and modulate host Th2 immune responses against helminth infections [12–14]. Notable among the compiled list were 39 peptidases, including 11 metalloproteases, 12 cysteine proteases, 10 serine proteases, and six aspartic proteases (Table S29). It is noteworthy that the secreted peptidases, such as the M12 ‘astacins’, S33 serine proteases, and C1 cysteine proteases, play key roles in tissue penetration and degradation during migration and feeding and/or immune evasion/modulation in many parasites, including *A. suum* [12]. In addition, we found 21 lectins (C-type), 11 cell-adhesion molecules (immunoglobulins), and seven ASP-like homologs (venom allergen proteins) among the *B. schroederi* E/S proteins (Table S29). C-type lectins (C-TLs) have been proven to participate in various immune processes of the host, including antigen uptake and presentation, cell adhesion, and T cell polarization [12,14,18]. Some *B. schroederi* C-TLs were predicted to share homology with mammalian CD23 (low IgE-afﬁnity receptor) or macrophage mannose receptor, similar to the results found for C-TLs in *A. suum* [12,13] and TES-32 and TES-70 in *T. canis* [19,20], and could therefore bind to host carbohydrates and mediate immune evasion by inhibiting the migration of host immune cells. We further noted that 15 of the 21 C-TLs were highly transcribed genes in *B. schroederi* during the parasitic period, including the L5 and adult stages (Table S29), which probably mirrors their immunomolecular roles in manipulating, blocking and/or evading immune responses in the host. Similar to the results from previous studies in *A. suum* [12] and *T. canis* [21], genes encoding cell adhesion molecules were also found to be abundant among the set of E/S proteins in *B. schroederi* (Table S29). Cell adhesion molecules are known as central mediators in the regulation of leukocyte trafficking and play roles in various cellular immunoreactions, such as lymphocyte maturation, activation, and costimulation, as well as the generation of specific immune responses [22,23]. Although the numbers of *B. schroederi* genes inferred to express cell adhesion molecules were comparable to those found in *A. suum* and *T. canis*, differences in categories and quantitative levels were found among these three ascaridoid species. The immunoglobulins predominated among the cell adhesion molecules in *B. schroederi*, in contrast with the repertoire of the immunoglobulins, integrins, and cadherins in *T. canis* [21]. We also identified five genes encoding venom allergen proteins among the set of *B. schroederi* E/S proteins. These cysteine-rich proteins were originally found in hookworms and are called activation-associated proteins or *Ancylostoma*-secreted proteins (ASPs) [18,24,25], whose functions are likely involved in blocking immune responses and blood clotting [24,25]. We failed to find significantly increased expression of these genes during infections *in vivo*. Instead, some genes that exhibited no obvious similarity to known ASP families but have been termed ASP-related genes (ASPRs) in the hookworms *Ancylostoma ceylanicum* and *Necator americanus* [26] were upregulated. Thus, similar to ASPs, ASPRs might play compensatory roles and thus comprise an important element in ascaridoid infection *in vivo*.

Other E/S molecules of *B. schroederi* that were predicted to interact with the host and/or play immunomodulatory roles include homologs of 10 thioredoxins, seven cathepsins, five cystatins, and five serpins (Table S29). Although there are some lifecycle similarities among ascaridoids, as demonstrated based on the nature and extent of molecules identified, it appears that the host immune responses against the parasitic stages of *B. schroederi* are distinct from those associated with *Ascaris* and *Toxocara*. Thus, more experimental confirmations are still needed. Combined, these findings suggest that *B. schroederi* has a substantial arsenal of ES proteins that are likely involved in host-parasite interactions and host immunomodulation and evasion.

**References**

[1] Chan PP, Lowe TM. GtRNAdb: a database of transfer RNA genes detected in genomic sequence. Nucleic Acids Res 2009;37:93–7.

[2] Iben JR, Maraia RJ. tRNAomics: tRNA gene copy number variation and codon use provide bioinformatic evidence of a new anticodon:codon wobble pair in a eukaryote. RNA 2012;18:1358–72.

[3] Fedyunin I, Lehnhardt L, Böhmer N, Kaufmann P, Zhang G, Ignatova Z. tRNA concentration fine tunes protein solubility. FEBS Lett 2012;586:3336–40.

[4] Lamichhane TN, Blewett NH, Crawford AK, Cherkasova VA, Iben JR, Begley TJ, et al. Lack of tRNA modification isopentenyl-A37 alters mRNA decoding and causes metabolic deficiencies in fission yeast. Mol Cell Biol 2013;33:2918–29.

[5] Tarr DE. Distribution and characteristics of ABFs, cecropins, nemapores, and lysozymes in nematodes. Dev Comp Immunol 2012;36:502–20.

[6] Willis C, Wang CK, Osman A, Simon A, Pickering D, Mulvenna J, et al. Insights into the membrane interactions of the saposin-like proteins Na-SLP-1 and Ac-SLP-1 from human and dog hookworm. PLoS One 2011;6:e25369.

[7] Dierking K, Yang W, Schulenburg H. Antimicrobial effectors in the nematode *Caenorhabditis elegans*: an outgroup to the Arthropoda. Philos Trans R Soc Lond B Biol Sci 2016;371:20150299.

[8] Lim MP, Firdaus-Raih M, Nathan S. Nematode peptides with host-directed anti-inflammatory activity rescue *Caenorhabditis elegans* from a *Burkholderia pseudomallei* infection. Front Microbiol 2016;7:1436.

[9] Han H, Wei W, Hu Y, Nie Y, Ji X, Yan L, et al. Diet evolution and habitat contraction of giant pandas via stable isotope analysis. Curr Biol 2019;29:664–9.

[10] Hu Y, Wu Q, Ma S, Ma T, Shan L, Wang X, et al. Comparative genomics reveals convergent evolution between the bamboo-eating giant and red pandas. Proc Natl Acad Sci U S A 2017;114:1081–6.

[11] Zhao H, Yang JR, Xu H, Zhang J. Pseudogenization of the umami taste receptor gene Tas1r1 in the giant panda coincided with its dietary switch to bamboo. Mol Biol Evol 2010;27:2669–73.

[12] Jex AR, Liu S, Li B, Young ND, Hall RS, Li Y, et al. *Ascaris suum* draft genome. Nature 2011;479:529–33.

[13] Hewitson JP, Grainger JR, Maizels RM. Helminth immunoregulation: The role of parasite secreted proteins in modulating host immunity. Mol Biochem Parasitol 2009;167:1–11.

[14] Harnett W. Secretory products of helminth parasites as immunomodulators. Mol Biochemic Parasitol 2014;195:130–6.

[15] McSorley HJ, Hewitson JP, Maizels, RM. Immunomodulation by helminth parasites: Defining mechanisms and mediators. Int J Parasitol 2013;43:301–10.

[16] Maizels RM. *Toxocara canis*: molecular basis of immune recognition and evasion. Vet Parasitol 2013;193:365–74.

[17] Maizels RM, Schabussova I, Callister DM, Nicoll G. Molecular Biology and Immunology of Toxocara canis. In: Holland CV, Smith HV, editors. Toxocara The Enigmatic Parasite, Cambridge: CABI Publishing; 2006, p. 3–17.

[18] Schwarz EM, Hu Y, Antoshechkin I, Miller MM, Sternberg PW, Aroian RV. The genome and transcriptome of the zoonotic hookworm *Ancylostoma ceylanicum* identify infection-specific gene families. Nat Genet 2015;47:416–22.

[19] Loukas A, Doedens A, Hintz M, Maizels RM. Identification of a new C-type lectin, TES-70, secreted by infective larvae of *Toxocara canis*, which binds to host ligands. Parasitology 2000;121:545–54.

[20] Maizels RM, Tetteh KK, Loukas A. *Toxocara canis*: genes expressed by the arrested infective larval stage of a parasitic nematode. Int J Parasitol 2000;30:495–508.

[21] Zhu XQ, Korhonen PK, Cai H, Young ND, Nejsum P, von Samson-Himmelstjerna G, et al. Genetic blueprint of the zoonotic pathogen *Toxocara canis*. Nat Commun 2015;6:6145.

[22] Figliuolo da Paz VR, Figueiredo-Vanzan D, Dos Santos Pyrrho A. Interaction and involvement of cellular adhesion molecules in the pathogenesis of *Schistosomiasis mansoni*. Immunol Lett 2019;206:11–8.

[23] Johansson MW. Cell adhesion molecules in invertebrate immunity. Dev Comp Immunol 1999;23:303–15.

[24] Cantacessi C, Campbell BE, Visser A, Geldhof P, Nolan MJ, Nisbet AJ, et al. A portrait of the "SCP/TAPS" proteins of eukaryotes -- developing a framework for fundamental research and biotechnological outcomes. Biotechnol Adv 2009;27:376–88.

[25] Osman A, Wang CK, Winter A, Loukas A, Tribolet L, Gasser RB, et al. Hookworm SCP/TAPS protein structure: a key to understanding host–parasite interactions and developing new interventions. Biotechnol Adv 2012;30:652–7.

[26] Tang YT, Gao X, Rosa BA, Abubucker S, Hallsworth-Pepin K, Martin J, et al. Genome of the human hookworm *Necator americanus*. Nat Genet 2014;46:261–9.


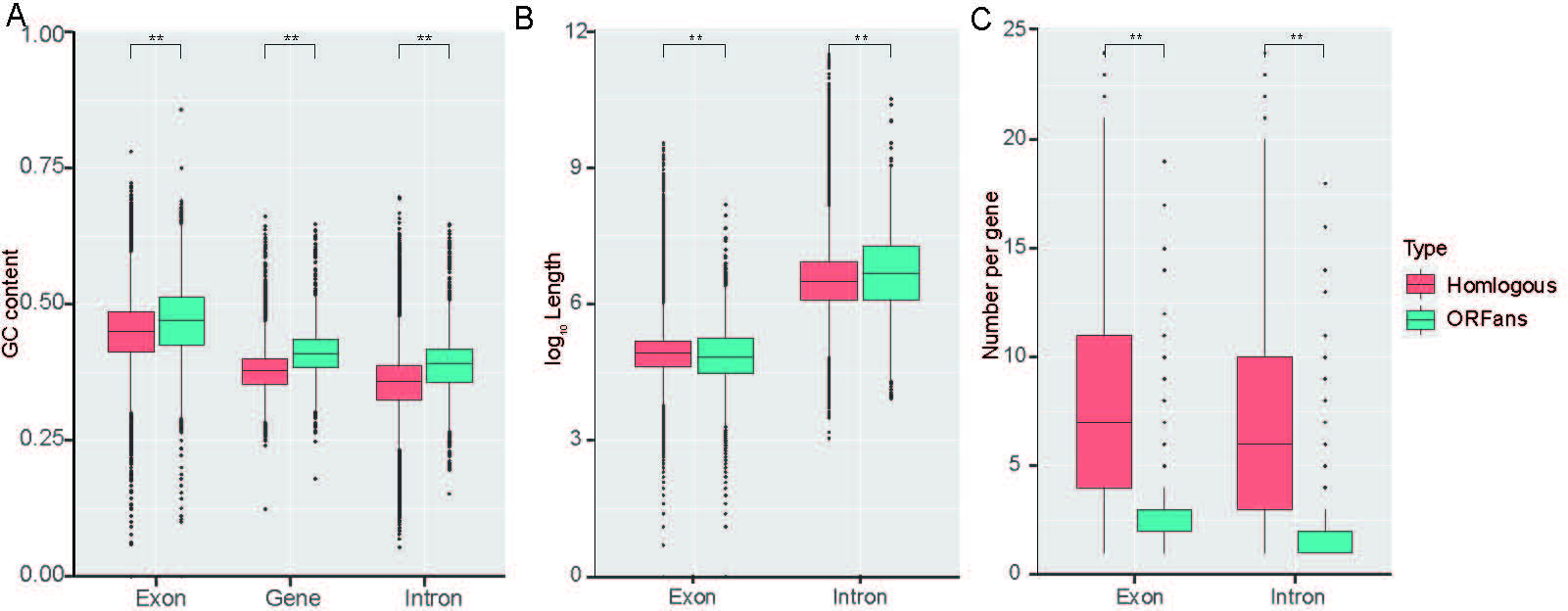


**Figure S14 Comparison between homologues and ORFans in *B. schroederi***

**A.** GC Content of gene, exon, and intron sequences. **B.** Length of exon and intron sequences. **C.** Exon and intron numbers for each gene. Statistically significant differences (*P* < 0.01) are indicated by double asterisks.


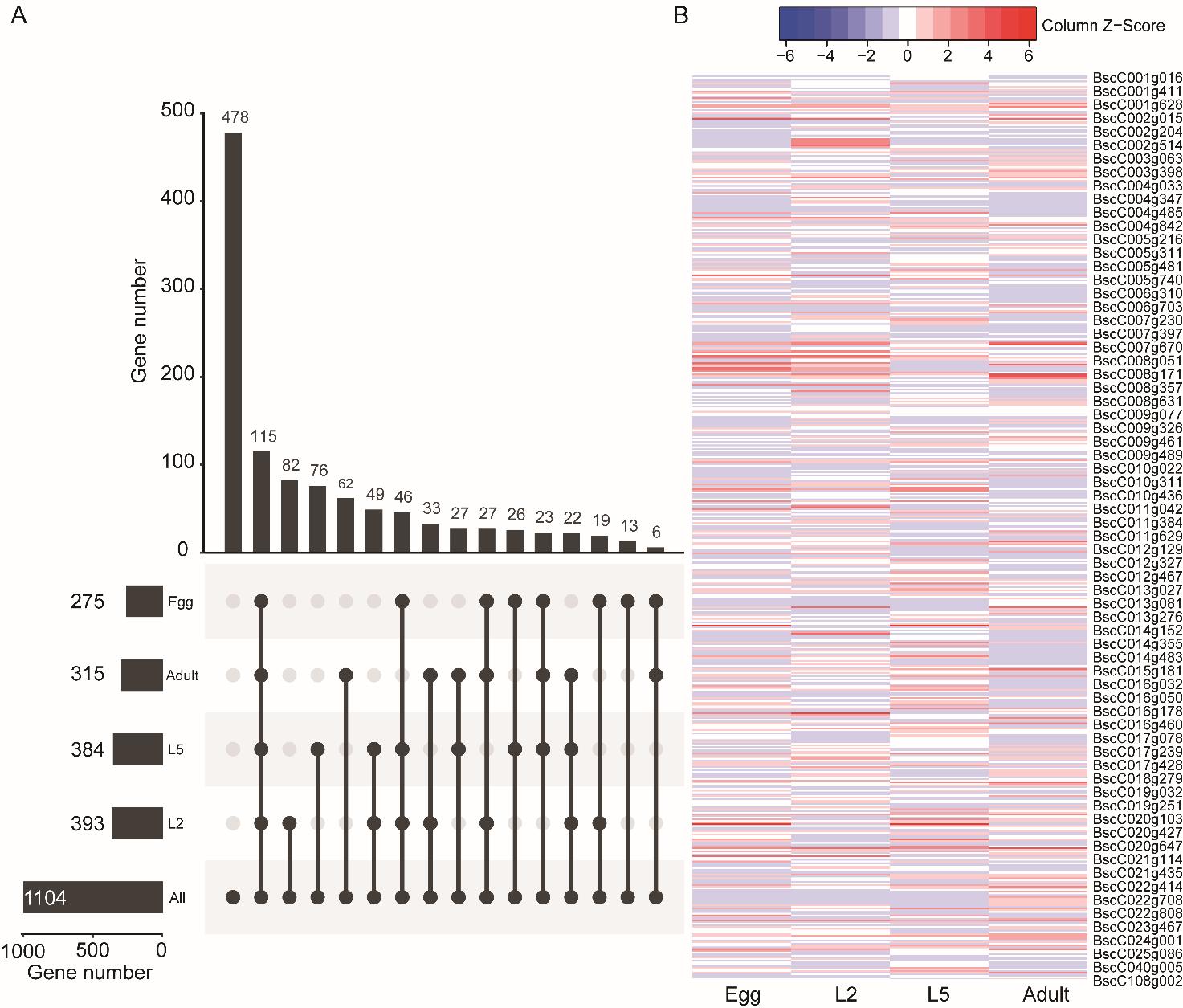


**Figure S15 Gene expression of ORFans in *B. schroederi***

**A.** Number of expressed genes in the four stages. **B.** Heatmap of ORFan gene expression.





**Figure S16 Expansion times of genes related to nutrition transporters in *B. schroederi***

**A.** ABC transporter. **B.** Sugar (and other) transporter. **C.** MFS/sugar transport protein. **D.** Neutral and basic amino acid transport protein. **E.** Transmembrane amino acid transporter protein. **F.** Long-chain fatty acid transport protein.





**Figure S17 Expansion times of enzyme genes related to nutrition metabolisms in *B. schroederi***

**A.** Hexokinase (EC:2.7.1.1). **B.** Dihydrolipoate acetyltransferase (EC:2.3.1.12). **C.** Putative citrate synthase (EC:2.3.3.1). **D.** Dehydrogenase E1 (EC:1.2.4.1). **E.** Glutamate dehydrogenase (EC:1.4.1.3). **F.** Asparagine synthetase (EC:6.3.5.4).
